# Microglia in the hypothalamus respond to tumor‐derived factors and are protective against cachexia during pancreatic cancer

**DOI:** 10.1002/glia.23796

**Published:** 2020-02-10

**Authors:** Kevin G. Burfeind, Xinxia Zhu, Mason A. Norgard, Peter R. Levasseur, Christian Huisman, Katherine A. Michaelis, Brennan Olson, Daniel L. Marks

**Affiliations:** ^1^ Papé Family Pediatric Research Institute Oregon Health & Science University Portland Oregon; ^2^ Medical Scientist Training Program, Oregon Health & Science University Portland Oregon; ^3^ Knight Cancer Institute Oregon Health & Science University Portland Oregon; ^4^ Brenden‐Colson Center for Pancreatic Care Oregon Health and & Science University Portland Oregon

**Keywords:** brain, cachexia, hypothalamus, microglia, neuroinflammation, pancreatic cancer

## Abstract

Microglia in the mediobasal hypothalamus (MBH) respond to inflammatory stimuli and metabolic perturbations to mediate body composition. This concept is well studied in the context of high fat diet induced obesity (HFDO), yet has not been investigated in the context of cachexia, a devastating metabolic syndrome characterized by anorexia, fatigue, and muscle catabolism. We show that microglia accumulate specifically in the MBH early in pancreatic ductal adenocarcinoma (PDAC)‐associated cachexia and assume an activated morphology. Furthermore, we observe astrogliosis in the MBH and hippocampus concurrent with cachexia initiation. We next show that circulating immune cells resembling macrophages infiltrate the MBH. PDAC‐derived factors induced microglia to express a transcriptional profile in vitro that was distinct from that induced by lipopolysaccharide (LPS). Microglia depletion through CSF1‐R antagonism resulted in accelerated cachexia onset and increased anorexia, fatigue, and muscle catabolism during PDAC. This corresponded with increased hypothalamic–pituitary–adrenal (HPA) axis activation. CSF1‐R antagonism had little effect on inflammatory response in the circulation, liver, or tumor. These findings demonstrate that microglia are protective against PDAC cachexia and provide mechanistic insight into this function.

## Graphical abstract

• Microgliosis occurs in the mediobasal hypothalamus early in pancreatic cancer.• Microglia respond to pancreatic cancer‐derived factors and produce arginase‐1.• Microglia depletion worsens cachexia during pancreatic cancer.

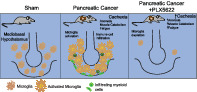

## 1 INTRODUCTION

Microglia, resident macrophages of the central nervous system (CNS), play a key role in regulating metabolic function via their activity within the MBH (García‐Cáceres et al., 2018). Microglia in this region sense various circulating metabolites and in turn influence neuronal activity (Béchade, Cantaut‐Belarif, & Bessis, 2013). The role of microglia in mediating metabolic dysfunction during obesity is well documented (Mendes, Kim, Velloso, & Araújo, 2018; Thaler et al., 2012; Valdearcos et al., 2017). Microgliosis in the MBH is a key feature of obesity, and attenuating microglial inflammatory activity can alleviate metabolic dysfunction and weight gain during diet‐induced obesity (Valdearcos et al., 2017).

While microglial activation and local production of inflammatory cytokines in the MBH contribute to weight gain and adipose accumulation during HFDO, the actions of cytokines in the MBH also mediate tissue *catabolism* during other chronic diseases (Burfeind, Michaelis, & Marks, 2016). Cachexia is a chronic disease‐associated metabolic syndrome consisting of anorexia, decreased locomotor activity, and tissue catabolism (Fearon et al., 2011). It accompanies several diseases, such as cancer, rheumatoid arthritis, and Alzheimer's disease (von Haehling, Anker, & Anker, 2016). There is abundant evidence showing that inflammation, particularly in the MBH, is associated with catabolism during chronic disease and can cause signs and symptoms similar to those observed in cachexia (Braun et al., 2011; Burfeind et al., 2016; Burfeind et al., 2018; Zhu et al., 2019).

Herein lies a paradox, where inflammation in the MBH is thought to drive both anabolism and catabolism. No studies assessed the role of microglia during cachexia. Moreover, the cellular source of inflammatory mediators in the MBH during cachexia is not known and remains a topic of active investigation. Therefore, we investigated the functions of microglia during cancer‐associated cachexia. We utilized a well‐characterized mouse model of pancreatic ductal adenocarcinoma (PDAC), a devastating neoplasm of the exocrine pancreas nearly always accompanied by cachexia (Bachmann et al., 2008). During PDAC, cachexia is associated with increased mortality (Bachmann et al., 2008), decreased quality of life (Fouladiun et al., 2007), and decreased tolerance to chemotherapy (Prado et al., 2009). Despite this serious clinical concern, there are currently no effective treatments for PDAC‐associated cachexia.

We observed that both microgliosis and astrogliosis occur early in a mouse model of PDAC‐associated cachexia. Next, we demonstrated the circulating myeloid cells infiltrate the MBH during PDAC. We then showed, using an ex vivo culture system, that microglia respond to tumor‐derived factors, including robust induction of arginase‐1 mRNA. Lastly, we observed that microglia depletion worsened cachexia, including increased anorexia, muscle catabolism, and fatigue. This was associated with increased HPA axis activation. These results show that microglia in the MBH are protective during PDAC cachexia and have a role in preserving metabolic function during cancer‐induced chronic systemic inflammation.

## 2 METHODS

### 2.1 Animals and CSF‐1R antagonist treatment

Male 20–25 g WT C57BL/6J (stock no. 000664) were purchased from Jackson Laboratories. Animals were aged between 7 and 12 weeks at the time of study and maintained at 27°C on a normal 12:12 hr light/dark cycle and provided ad libitum access to water and food. Experiments were conducted in accordance with the National Institutes of Health Guide for the Care and Use of Laboratory Animals, and approved by the Animal Care and Use Committee of Oregon Health & Science University.

PLX5622 was generously provided by Plexxikon Inc. (Berkeley, CA). This is an orally active CSF‐1R antagonist previously shown to deplete microglia (Huang et al., 2018). PLX5622 was incorporated into chow by Research Diets Inc. (New Brunswick, NJ). The same chow (AIN chow) without PLX5622 was provided as a control. Animals were placed on PLX5622‐containing chow or AIN chow starting 6 days prior to tumor inoculation.

### 2.2 KPC cancer cachexia model

Our lab generated a mouse model of PDAC‐associated cachexia by a single intraperitoneal (IP) injection of murine‐derived C57BL/6 KRAS^G12D^ P53^R172H^ Pdx‐Cre^+/+^ (KPC) pancreatic ductal adenocarcinoma (PDAC) (Michaelis et al., 2017). These cells are derived from tumors in C57BL/6 mice heterozygous for oncogenic KRAS^G12D^ and point mutant TP53^R172H^ (both downstream of lox‐stop‐lox cassettes) with expression induced and targeted to the pancreas via the PDX‐1‐Cre driver (Foley et al., 2015). Cells were maintained in RPMI supplemented with 10% heat‐inactivated FBS, and 50 U/ml penicillin/streptomycin (Gibco, Thermofisher), in incubators maintained at 37°C and 5% CO_2_. In the week prior to tumor implantation, animals were transitioned to individual housing to acclimate to experimental conditions. Animal food intake and body weight were measured once daily at 9 a.m. Sham‐operated animals received PBS in the same volume. Voluntary home cage locomotor activity was measured via MiniMitter tracking devices (Respironics). Mice were implanted 6 days prior to tumor implantation with MiniMitter transponders in the intrascapular subcutaneous space. Using these devices, movement counts in *x*‐axis, *y*‐axis, and *z*‐axis were recorded in 5 min intervals (Vital View, MiniMitter). Only dark cycle activity was analyzed, as our previous studies demonstrated little movement during the light phase (Michaelis et al., 2017).

Animals were euthanized between 7 and 10 days post inoculation, when food intake was consistently decreased and locomotor activity was visibly reduced, yet signs of end‐stage disease (ascites, unkempt fur, hypothermia, etc.) were not present (Michaelis et al., 2017).

### 2.3 Generation of Ly5.1‐EGFP chimera mice

WT C57BL/6J male mice aged 8–10 weeks were injected IP with the alkylating agent treosulfan (Ovastat®, a generous gift from Joachim Baumgart at Medac GmbH, Germany) at a dose of 1,500 mg/kg/day for three consecutive days prior to the day of bone marrow transplant (BMT). Twenty four hours after the third treosulfan injection, a Ly5.1‐EGFP male or female donor mouse aged between 2–6 months was euthanized and femurs, tibias, and humeri were dissected. After muscle and connective tissue were removed, marrow cells were harvested by flushing the marrow cavity of dissected bones using a 25‐gauge needle with Iscove's modified Dulbecco's medium supplemented with 2% FBS. The harvested cells were treated with RBC lysis buffer, filtered with a 70 μm cell strainer, and counted. 3 × 10^6^ cells in 200 μl HBSS were transplanted immediately into each recipient mouse via tail vein injection. To prevent infection during an immunocompromised period, recipient mice received amoxicillin dissolved in their drinking water (150 mg/L) for 2 weeks starting on the first day of treosulfan injection. GFP BMT mice were given at least 8 weeks for marrow reconstitution and recovery.

### 2.4 Immunofluorescence immunohistochemistry

Mice were anesthetized using a ketamine/xylazine/acepromazine cocktail and sacrificed by transcardial perfusion fixation with 15 ml ice cold 0.01 M PBS followed by 25 ml 4% paraformaldehyde (PFA) in 0.01 M PBS. Brains were post‐fixed in 4% PFA overnight at 4°C and cryoprotected in 20% sucrose for 24 hr at 4°C before being stored at −80°C until used for immunohistochemistry. Immunofluorescence immunohistochemistry was performed as described below. Thirty micrometer free‐floating coronal sections were cut from perfused brains using a Leica sliding microtome. Sections were incubated for 30 min at room temperature in blocking reagent (5% normal donkey serum in 0.01 M PBS and 0.3% Triton X‐100). After the initial blocking step, sections were incubated in primary antibody in blocking reagent for 24 hr at 4°C, followed by incubation in secondary antibody (also listed below) for 2 hr at 4°C. Between each stage, sections were washed thoroughly with 0.01 M PBS. Sections were mounted onto gelatin‐coated slides and coverslipped using Prolong Gold antifade media with DAPI (Thermofisher).

For primary antibodies, rabbit anti‐Iba‐1 (Wako, NCNP24, 1:1000), mouse anti NeuN (EMD Millipore, clone A60, 1:1000), and mouse anti‐GFAP (Millipore, GA5, 1:1000). The following secondary antibodies were used, all derived from donkey: anti‐rabbit AF488 (1:500), anti‐mouse AF633 (1:500), and anti‐rabbit AF555 (1:1000), anti‐goat AF488 (1:500).

### 2.5 Image acquisition and analysis

All images were acquired using a Nikon confocal microscope. Three 7‐layer flattened Z‐stack images of the hippocampus, MBH, and cortex were acquired using a 20× objective. Images were 2048 × 2048 pixels, with a pixel size of 0.315 μm. The cortex was defined as the field of view immediately dorsal the cingulum and directly lateral to the midline, also dorsal to the corpus collusum. The hippocampus was identified by the granule cell layer of the dentate gyrus, which was positioned at the left end of each image. The MBH was defined as the region surrounding the third ventricle at the base of the brain, starting rostrally at the end of the optic chiasm when the arcuate nucleus appears (−1.22 mm from bregma) and ending caudally at the mammillary body (−2.70 mm from bregma). Within the MBH, the arcuate nucleus (ARC) was defined as the regions to the left and right of the third ventricle, and the median eminence (ME) was defined as the semicircular extension of tissue directly below the third ventricle.

Microglia and astrocyte morphology in the MBH and hippocampus were quantified using Fiji (ImageJ, NIH). Microglia morphology was also quantified in the cortex, but we did not perform astrocyte morphology analysis in this region due to lack of GFAP immunoreactivity in tumor and sham groups. Images were uploaded by a blinded reviewer (KGB) and converted to 8‐bit greyscale images. After thresholding, microglia and astrocytes were identified using the “analyze particle” function, which measured cell number (for microglia), mean GFAP intensity (for astrocytes), and percent area covered by Iba‐1 or GFAP staining.

Quantification of infiltrating immune cells in the ME and ARC was performed by blind‐counting the number GFP+ cells in comparable sections of sham and tumor mice (*n* = 3, 3 sections per mouse).

Neuron quantification in the arcuate nucleus was performed by counting the number of NeuN positive cells using the Fiji cell counter plugin.

### 2.6 Quantitative real‐time PCR

For in vivo experiments, mice were euthanized with a lethal dose of a ketamine/xylazine/acepromazine. Hypothalamic blocks were dissected, snap frozen, and stored in −80°C until analysis. For both in vivo and in vitro experiments, RNA was extracted using an RNeasy mini kit (Qiagen) according to the manufacturer's instructions. cDNA was transcribed using TaqMan reverse transcription reagents and random hexamers according to the manufacturer's instructions. PCR reactions were run on an ABI 7300 (Applied Biosystems), using TaqMan universal PCR master mix with the following TaqMan mouse gene expression assays: *18 s* (Mm04277571_s1), *Tnf* (Mm00443258_m1), *Il‐6* (Mm01210732_g1), *Il‐1b* (Mm00434228_m1), *Il‐10* (Mm01288386_m1), *Tgfb* (Mm01227699_m1), *Arg1* (MM00475988_m1), *Ido1* (Mm00492590_m1), *Nos2* (Mm00440502_m1), *Tmem119* (Mm0052305_m1), *Igf‐1* (Mm00439560_m1), *Crp* (MM00432680_g1), *Cd68* (Mm03047343_m1), *Crh* (Mm01293920_s1), *Kiss1* (Mm03058560_m1), *Gnrh* (Mm01315604_m1), *Selp* (Mm00441297_m1), *Vcam‐1* (Mm01320970_m1), and *Icam‐1* (Mm00516023_m1).

Relative expression was calculated using the ΔΔCt method and normalized to WT vehicle treated, sham control, or 7 days post inoculation AIN control chow. Statistical analysis was performed on the normally distributed ΔCt values.

### 2.7 Primary microglia culture and KPC‐conditioned media treatment

Primary mixed‐glial cultures containing microglia and astrocytes were prepared from neonatal mouse cortices. Brain cortices from 1–3 day old newborn mouse pups were dissected, freed of the meninges, and then digested with papain (Worthington Biochemical Corporation). The mixed cortical cells were passed through a 70‐μm cell strainer and seeded in 75 cm^2^ flasks in DMEM media (low glucose with l‐glutamine, 10% FBS and 1% penicillin/streptomycin). Media was refreshed every 3–4 days for 9–16 days. Microglia were isolated by shaking flasks at 200 rpm at 37°C for 1 hr. Cells were re‐plated into 6 well plates at 5 × 10^5^/well and maintained in DMEM media for 24 hr before stimulation. More than 90% of these isolated cells were confirmed as microglia by Iba1 staining and flow cytometry (CD45 + CD11b+ cells, data not shown).

KPC tumor cells were cultured in a 75‐cm^2^ flask until confluent. Twenty four hours prior to treatment, 13 ml fresh media (RPMI supplemented with 10% FBS and 1% penicillin–streptomycin) was added for generating KPC‐conditioned media. On the treatment day, 4 ml KPC‐conditioned media mixed with 1 ml fresh RPMI media (to ensure treated microglia were not nutrient starved) was added to each of the three wells of the 6 well plate containing microglia. The other three wells each received 5 ml control media (RPMI media). Three additional wells received 5 ml control media containing 10 ng/ml LPS. Sixteen hours after treatment, media was removed, and adherent cells were washed with PBS then lysed. RNA was then extracted from cell lysate using a Qiagen RNAEasy kit.

### 2.8 Flow cytometry

Mice were anesthetized using a ketamine/xylazine/acepromazine cocktail and perfused with 15 ml ice cold 0.01 M PBS to remove circulating leukocytes. If circulating leukocytes were analyzed, blood was collected prior to perfusion via cardiac puncture using a 25‐gauge needle syringe, then placed in an EDTA coated tube. After perfusion, organs were extracted and immune cells were isolated using the following protocols:

#### 2.8.1 Liver

Livers were pushed through a 70 μm nylon strainer, and then washed once with RPMI. The resulting suspension was resuspended in a 40 ml digestion solution containing 1 mg/ml type II collagenase (Sigma) and 1% DNAse (Sigma) in RPMI, then placed in a 37°C incubator for 1 hr. After digestion, the suspension was placed on ice for 5 min, then the top 35 ml was collected and centrifuged. The resulting pellet was resuspended in 10 ml 35% percoll and centrifuged to remove debris, then treated with RBC lysis buffer. The resulting cell suspension was washed with RPMI, then cells were incubated in 100 μl of PBS containing antibodies for 30 min, then washed with RPMI.

#### 2.8.2 Tumor

A 0.2–0.3 g piece of pancreatic tumor was removed, minced in a digestion solution containing 1 mg/ml type II collagenase and 1% DNAse in RPMI, and then placed in a 37°C incubator for 1 hr. After digestion, cells were washed with RPMI, then incubated in 100 μl of PBS containing antibodies for 30 min at 4°C. Cells were then washed once with RPMI.

#### 2.8.3 Blood

150 μl of blood was collected via cardiac puncture with a 25‐gauge needle syringe. Red blood cells were then lysed with 1X RBC lysis buffer. The resulting cell suspension was washed with RPMI, then cells were incubated in 100 μl of PBS containing antibodies for 30 min at 4°C, then washed with RPMI.

#### 2.8.4 Gating strategy

Cells were gated on LD, SSC singlet, and FSC singlet. Immune cells were defined as CD45+ cells. Leukocytes were identified as either myeloid cells (CD45 + CD11b+) or lymphocytes (CD45 + CD11b−). From myeloid cells Ly6C^low^ monocytes (Ly6C^low^Ly6G−), Ly6C^mid^ monocytes (Ly6C^mid^Ly6G−, only observed in the blood) Ly6C^high^ monocytes (Ly6C^high^Ly6G−), and neutrophils (Ly6C^mid/high^Ly6G+) were identified. In tumor tissue, myeloid cells were gated as either Ly6G+ neutrophils or Ly6G− myeloid cells (“other myeloid cells”). Ly6G− myeloid cells were further phenotyped as F4/80+ tumor associated macrophages or F4/80‐Ly6C+ inflammatory monocytes in the tumor. In the liver, myeloid cells were phenotyped as F4/80+ macrophages or F4/80− non‐macrophages. F4/80− non‐macrophages were further phenotype as Ly6C^hi^Ly6G− inflammatory monocytes or Ly6G+ neutrophils. From lymphocytes, CD3+ cells were identified as T‐cells.

#### 2.8.5 Antibodies

All antibodies were purchased from BioLegend, except for Live/Dead, which was purchased from Invitrogen (Fixable Aqua, used at 1:200 dilution). The following anti‐mouse antibodies were used, with clone, fluorophore, and dilution indicated in parenthesis: CD3 (17A2, PE, 1:100), CD11b (M1/70, FITC, 1:200), CD45 (30‐F11, PerCP/Cy5.5, 1:400), Ly6C (HK1.4, PerCP, 1:100), Ly6G (1A8, PE/Cy7, 1:800), F4/80 (BM8, APC, 1:100), F4/80 (BM8, BV785, 1:100), CD206 (MMR, APC, 1:100).

### 2.9 Serum corticosterone ELISA assay

Plasma corticosterone concentrations were determined using DetectX enzyme‐linked immunosorbent assay (ELISA) kit (Arbor Assays, Catalog number KO14‐H1), according to the manufacturer's instruction. The kit measures total corticosterone in plasma including the corticosterone combined with corticosteroid‐binding globulin (CBG). After 1:100 dilution, assay values of all samples were within the standard curve with a detectable range from 10,000 to 78.125 pg/ml.

### 2.10 Statistical analysis

Data are expressed as means ± *SEM*. Statistical analysis was performed with Prism 7.0 software (Graphpad Software Corp). When two groups were compared, data were analyzed with Student's *t*‐test. When two or more groups were compared, One‐way (when multiple groups were compared to a single sham group) or Two‐way (when there were multiple genotypes within tumor and sham groups being compared) ANOVA analysis. For single time point experiments, the two factors in ANOVA analysis were genotype or treatment. In repeated measures experiments, the two factors were group and time. Main effects of genotype, treatment, group, and/or time were first analyzed, and if one effect was significant, Bonferroni post hoc analysis was then performed. For all analyses, significance was assigned at the level of *p* < .05.

## 3 RESULTS

### 3.1 Hypothalamic gliosis occurs early during PDAC cachexia

**Figure 1:**
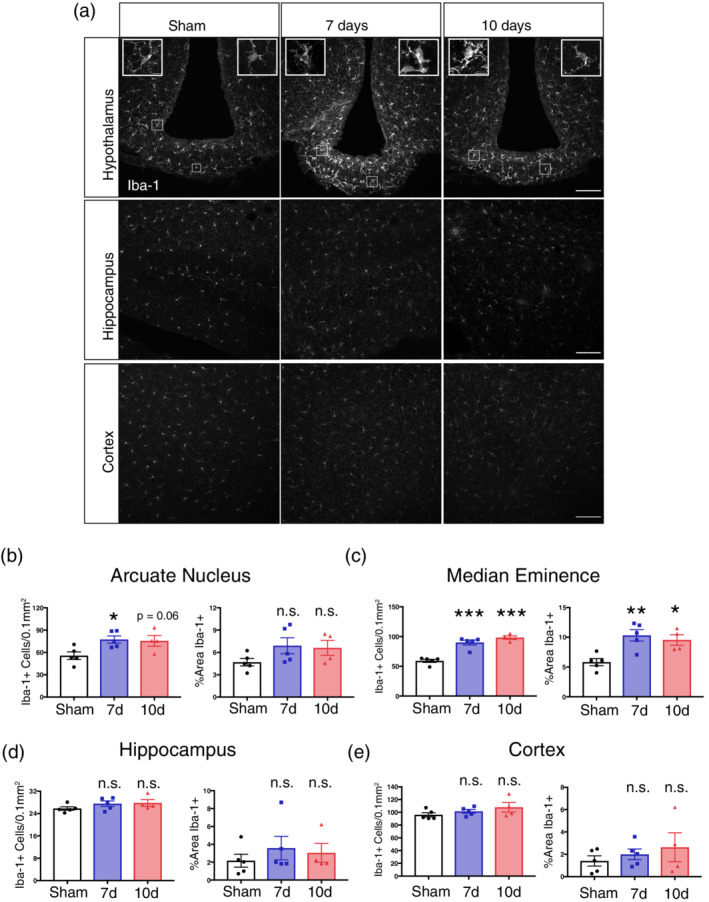
Microgliosis occurs in the MBH throughout PDAC cachexia. (a) Representative confocal microscopy images of Iba‐1 immunoreactivity within the MBH, hippocampus, and cortex from either a sham, 7 dpi, or 10 dpi mouse brain. Scale bar = 100 μm. Top left inset = arcuate nucleus microglia within left box. Top right inset = median eminence microglia within right box. Inset scale bars = 10 μm. B‐E) Analysis of microglia number (left) or percent area Iba‐1+ (right) in the arcuate nucleus, median eminence, hippocampus, or cortex. n.s. = not significant, ***p < .001, **p < .01, *p < .05 compared to sham in one‐way ANOVA analysis [Color figure can be viewed at wileyonlinelibrary.com]

We first assessed whether microgliosis occurred in different brain regions in our PDAC cachexia mouse model. This model is generated through a single intraperitoneal (IP) injection of KPC PDAC cells. It is extensively characterized (Burfeind et al., 2018; Michaelis et al., 2017; Michaelis et al., 2019; Zhu et al., 2019) and recapitulates all of the key signs and symptoms of cachexia seen in humans, including anorexia, muscle catabolism, fatigue, and loss of fat mass (Michaelis et al., 2017). After a single IP injection, these animals develop tumors almost exclusively in the pancreas and do not develop peritoneal disease until terminal stage (>14 dpi) (Michaelis et al., 2017). We used Iba‐1 to label brain microglia, and assessed morphology and number at 7 days post inoculation (dpi), a point when the animals begin to develop muscle catabolism, anorexia, and decreased locomotor activity, and 10 dpi, when all animals are cachectic yet still 3–4 days away from end‐stage signs (ascites, hypothermia, etc.). We observed increased microglia number during PDAC in the ME and ARC of the hypothalamus at 7 dpi and continuing at 10 dpi (Figure 1a–c). We also observed that microglia assumed an “activated” morphology, with retracted processes and increased soma size. This was evident in the ARC but was striking in the ME (insets in Figure 1a–c). Interestingly, microglia activation only occurred in the hypothalamus, with no increase in microglia number or Iba‐1 immunoreactivity in the hippocampus or cortex throughout the course of PDAC cachexia (Figure 1a,e).

### 3.2 Astrogliosis occurs early in PDAC cachexia

Next, we assessed whether astrocytes also assumed an activated morphology during PDAC cachexia. Using the same experimental setup described for microglia above, we used GFAP labeling to analyze astrocyte morphology in the hippocampus and hypothalamus at 7 and 10 dpi. We were unable to detect GFAP+ astrocytes within the cortex and it was therefore excluded from analysis. Similar to our findings for microglia, we observed astrocytosis in the MBH beginning at 7 dpi and becoming even more most robust at 10 dpi (Figure S1a–c) Unlike our findings for microglia, however, astrogliosis was just as robust in the hippocampus, with mean GFAP fluorescent intensity increased at 7 and 10 dpi (Figure S1a,d) While percent area covered by GFAP was not significantly increased in the hippocampus at either 7 or 10 dpi, a trend was observable (Figure S1d).

### 3.3 Circulating immune cells infiltrate the MBH during PDAC cachexia

**Figure 2:**
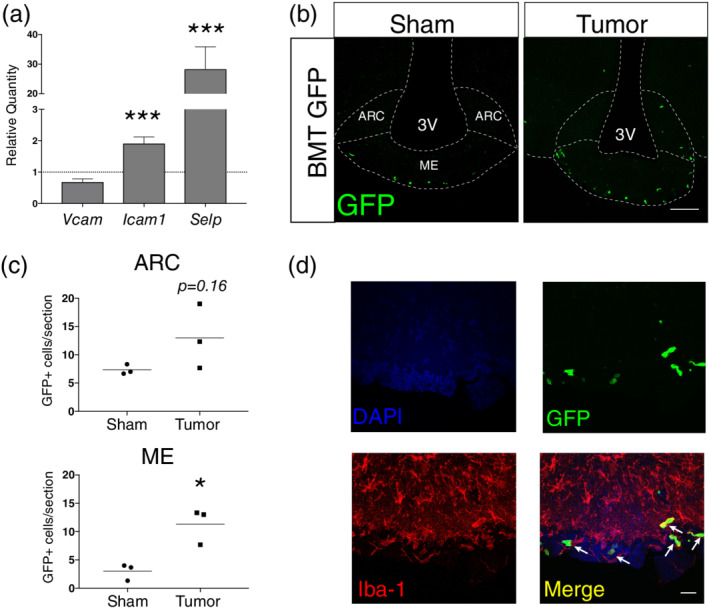
Circulating immune cells infiltrate the MBH during PDAC. a) qRT‐PCR analysis for leukocyte adhesion molecule transcripts in RNA extracted from whole hypothalamic at 10 dpi. Results are relative to sham. ***p < .001 relative to sham comparing ΔCt values in repeated measures one‐way ANOVA. Results consist of two independent experiments combined. *n* = 4–8/group. (b) Representative 20X confocal images MBH from sham and tumor (10 dpi) mice with median eminence and arcuate nucleus labeled on sham section. ME = median eminence. ARC = arcuate nucleus. Scale bar = 100 μm. (c) Quantification of B. *p < .05 compared to sham in *t*‐test. *n* = 3/group. (d) Representative 60× image of ME from BMT GFP tumor mouse, at 10 dpi. Scale bar = 20 μm. Arrows = GFP + Iba‐1+ infiltrating macrophages [Color figure can be viewed at wileyonlinelibrary.com]

Previous studies demonstrate the circulating myeloid cells infiltrate the MBH during HFDO, many of which express Iba‐1 (Valdearcos et al., 2017). Since we observed an increased number of Iba‐1+ cells in the ARC and ME during PDAC cachexia, we sought to determine whether these cells were infiltrating immune cells, or a product of microglia expansion. To answer this question, we first performed qRT‐PCR for leukocyte adhesion molecule transcripts on whole hypothalami at 10 dpi to determine if the machinery was in place for immune cell extravasation from the circulation into tissue. We dissected hypothalamic blocks from tumor‐bearing and sham animals and extracted RNA for qRT‐PCR. We chose to limit our analysis to a single time point (10 dpi) when all animals reliably developed cachexia and show upregulation of inflammatory cytokine transcripts in the MBH (Burfeind et al., 2018; Michaelis et al., 2017). We observed increased expression of *Icam1* (which codes for the ICAM‐1) and *Selp* (which codes for P‐selectin) in PDAC‐bearing animals compared to sham animals. (Figure 2a). Next, in order to determine if circulating immune cells infiltrate the MBH during PDAC, we generated GFP+ bone marrow chimera mice through conditioning WT mice with treosulfan to ablate marrow, followed by transplanting marrow from pan‐GFP mice (Ly5.1^GFP^). This system is advantageous because, unlike other alkylating agents, treosulfan does not cross or disrupt the blood brain barrier (Capotondo et al., 2012). Using this protocol, we regularly observe at least 75% chimerism (data not shown). We observed that GFP+ circulating immune cells infiltrate the MBH during PDAC at 10 dpi (Figure 2b). While there was a trend toward significance in the ARC, there were significantly more GFP+ cells in the ME in tumor‐bearing animals compared to sham animals (Figure 2c). Similar to studies on HFDO, we found that most GFP+ cells in the ME were Iba‐1+, and resembled macrophages (Figure 2d).

### 3.4 Microglia respond to PDAC tumor‐derived factors and produce arginase‐1 both in vivo and ex vivo

**Figure 3:**
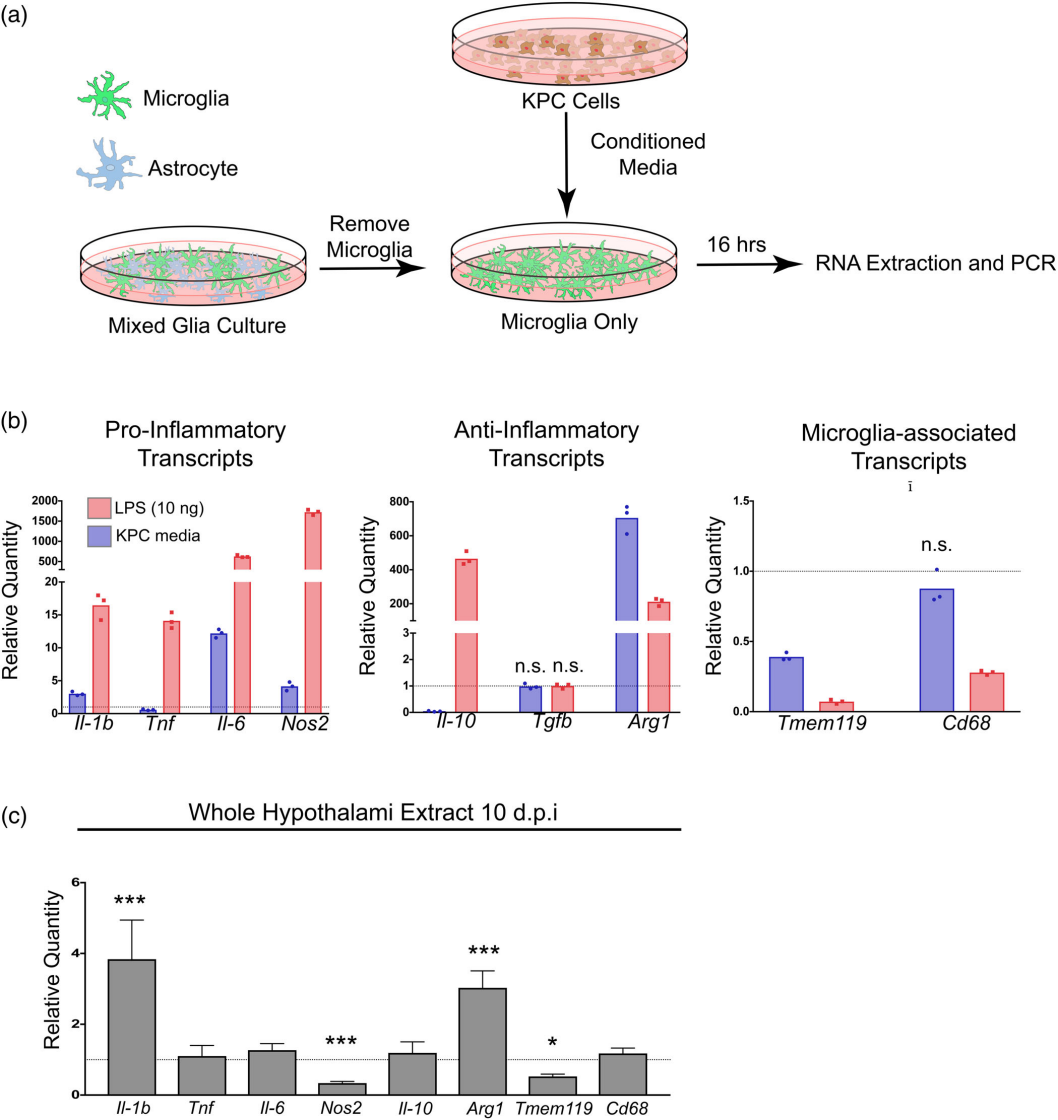
PDAC induces inflammatory mediator expression in vitro and in vivo. (a) Schematic representation of ex vivo KPC‐conditioned media treatment system. (b) qRT‐PCR analysis of pro‐inflammatory, anti‐inflammatory, and microglia‐associated transcript from primary microglia treated with either KPC‐conditioned media or 10 ng LPS. Values are relative to those from control media‐treated primary microglia. All comparisons at least *p* < .01 compared to control media‐treated in repeated measures one‐way ANOVA unless indicated otherwise. n.s. = not significant. *n* = 3/group. Results are representative of two independent experiments. (c) qRT‐PCR analysis of the same transcripts as B (except *Tgfb*) in RNA extracted from whole hypothalami. Results are relative to sham. ***p < .001, *p < .05 relative to sham comparing ΔCt values in repeated measures one‐way ANOVA. Results consist of two independent experiments combined. *n* = 4–8/group [Color figure can be viewed at wileyonlinelibrary.com]

Since we observed microglia activation during PDAC in vivo, we hypothesized that microglia would respond to PDAC‐derived factors in vitro. We utilized an ex vivo culture system in which isolated primary microglia were exposed to KPC‐conditioned media. We isolated mixed glia from one to three‐day old mouse pups, then after 14–16 days we removed microglia through shaking and treated them with KPC‐conditioned media (Figure 3a). Compared to the positive control (10 ng LPS), we observed minimal upregulation of the pro‐inflammatory transcripts *Il‐1b*, *Tnf*, *IL‐6*, and *Nos2* by KPC‐conditioned media (Figure 3b). Alternatively, we observed robust upregulation of the anti‐inflammatory transcript *Arg1* (coding for the enzyme arginase 1). While LPS induced a substantial upregulation of the anti‐inflammatory cytokine *Il‐10* (465‐fold increase), KPC‐conditioned media treatment resulted in robust downregulation of this transcript (40‐fold decrease). Neither LPS nor KPC‐conditioned media caused any change in expression of the anti‐inflammatory transcript *Tgfb*. Lastly, we assessed expression of transcripts previously identified as markers microglia activation (Orihuela, McPherson, & Harry, 2016). We observed a robust decrease in the transcript *Tmem119* in both LPS‐ and KPC‐ conditioned media‐treated microglia (indicative of microglia activation) (Figure 3b). Lastly, we assessed whether *Ido1*, which codes for IDO‐1, an immunoregulatory enzyme induced in macrophages by tumor‐derived factors (Zhao et al., 2012), was induced by KPC‐conditioned media. We did not observe any Ido1 expression in control‐ or KPC‐conditioned media treated microglia.

We next assessed whether these transcripts were differentially regulated during PDAC in vivo at 10 dpi. Similar to what occurred in KPC‐conditioned media treated primary microglia, we observed increased expression of *Il‐1b* and *Arg1* in the hypothalami of tumor‐bearing animals compared to sham animals, whereas there was decreased expression of *Nos2* and *Tmem119* (Figure 3c). There was no change in *Tnf*, *Il‐6*, *Il‐10*, or *Cd68* in PDAC mice. Similar to primary microglia, we observed minimal Ido1 expression in hypothalami of tumor‐bearing and sham animals (data not shown).

### 3.5 PLX5622 treatment worsens anorexia and decreases locomotor activity during PDAC

**Figure 4:**
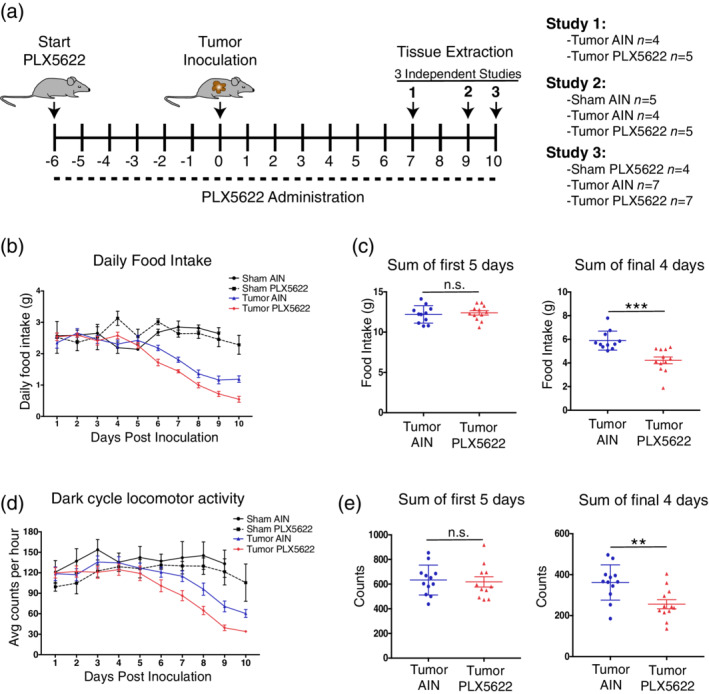
Microglia depletion worsens anorexia and decreases locomotor activity during PDAC. (a) Schematic of PLX5622 treatment protocol and tumor inoculation. Diagram depicts three independent studies investigating various stages of cachexia. Numbers listed on number line are in relation to tumor inoculation. Day 0 = tumor inoculation day. (b) Daily food intake after tumor inoculation, with two sham groups shown for reference. AIN = control chow. For food intake analysis, study 2 and 3 were combined. (c) Total food intake during the first 5 days of the study (pre‐cachexia stage) and final 4 days of the study (cachexia stage). n.s. = not significant, ***p < .001 for Student's *t*‐test. (d) Dark cycle home cage locomotor activity after tumor inoculation, with two sham groups shown for reference. Data shown are study 2 and 3 pooled. (e) Sum of average hourly counts for the first 5 days of the study (pre‐cachexia stage) and final 4 days of the study (cachexia stage). n.s. = not significant, **p < .01 in Student's *t*‐test [Color figure can be viewed at wileyonlinelibrary.com]

Based on our observation that PDAC induces microglia activation both in vitro and in vivo, we hypothesized that microglia influence cachexia symptoms. To test this hypothesis, we utilized the oral CSF1R inhibitor PLX5622, developed by Plexikkon, which depletes 75% of microglia within 3 days of administration, and 99% by 7 days (Huang et al., 2018). We conducted three independent studies in which we treated animals with PLX5622‐containing chow or control for 6 days then inoculated animals with KPC tumor cells IP. Animals were maintained on PLX5622 or control chow for the duration of the study, and euthanized at either 7, 9, or 10 dpi (Figure 4a). In the 6 days prior to tumor inoculation, PLX5622 did not induce any changes in food intake, body weight, or locomotor activity (Figure S2). Using PLX5622, we were able to achieve approximately 80% depletion of microglia in the MBH at 7 and 10 dpi (Figure S3a). Interestingly, we observed that microglia depletion by PLX5622 worsened cachexia, as evidenced by increased anorexia compared to untreated tumor‐bearing animals, beginning at 6 dpi (Figure 4b,c). There was no difference in food intake between groups prior to cachexia development (first 5 days, Figure 4c). PLX5622 treatment also was associated with decreased home cage locomotor activity in tumor‐bearing animals compared to untreated animals in during cachexia (final 4 days of the study, Figure 4d,e).

### 3.6 PLX5622 treatment worsens muscle catabolism and is associated with increased neuroendocrine activity during PDAC

**Figure 5:**
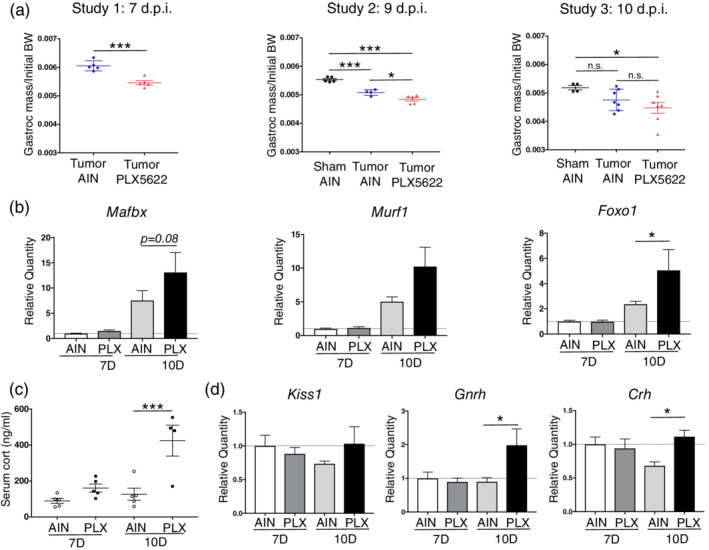
Microglia depletion worsens muscle catabolism and is associated with increased HPA axis activity during PDAC. (a) Muscle catabolism, determined by gastrocnemius mass normalized to initial body weight. Studies were not pooled since normalized muscle mass varied between study. n.s. = not significant. For study 1 (as described in Figure 4), ***p < .001 in Student's *t*‐test. For studies 2 and 3, ***p < .001, *p < .05 in one‐way ANOVA analysis. (b) qRT‐PCR analysis of *Mafbx*, *Murf1*, and *Foxo1* from RNA extracted from gastrocnemii. Values normalized to those from AIN 7d. PLX = PLX5622. *n* = 4–5/group. *p < .05 relative to AIN 10d comparing ΔCt values in Bonferroni post hoc analysis in two‐way ANOVA. (c) Serum corticosterone, measured by ELISA, at 7 and 10 dpi in PLX5622‐ and AIN‐treated tumor bearing mice. ***p < .001 in Bonferroni post hoc analysis in two‐way ANOVA. Cort = corticosterone. *n* = 4–5/group. (d) qRT‐PCR analysis of *Kiss1*, *Gnrh*, and *Crh* from RNA extracted from hypothalamic blocks at 7 and 10 dpi. Values normalized to those from AIN 7d. *p < .05 relative to AIN 10d comparing dCT values in Bonferroni post hoc analysis in two‐way ANOVA. *n* = 4–5/group [Color figure can be viewed at wileyonlinelibrary.com]

We previously demonstrated that muscle catabolism during cancer cachexia is induced by glucocorticoid production driven by activation of the HPA axis (Braun et al., 2011). Since we observed increased anorexia and decreased locomotor activity in PLX5622‐treated tumor‐bearing animals, we hypothesized that there would also be increased muscle catabolism and HPA axis activity. In all three studies described above, we observed that tumor‐bearing animals treated with PLX5622 had decreased gastrocnemius mass compared to that of untreated tumor‐bearing animals (Figure 5a). This corresponded with increased expression of the proteolytic enzymes *Mafbx* and *Murf1*, as well as the transcription factor *Foxo1*, which is important for muscle catabolism (T. P. Braun et al., 2011) (Figure 5b). To assess HPA axis activity, we measured serum corticosterone at 7 and 10 dpi in AIN‐ and PLX5622‐treated tumor‐bearing mice. We observed that at 10 dpi, PLX5622‐treated animals had significantly higher levels of circulating cortisol compared to AIN‐treated animals (Figure 5c), indicative of increased HPA axis activity. We next used qRT‐PCR to measure expression of transcripts associated with hypothalamic neuroendocrine activity. As expected, we observed a slight decrease in expression of *Crh* (coding for corticotrophin‐releasing hormone) from 7 to 10 dpi in AIN‐treated tumor‐bearing animals, which corresponds to negative feedback from corticosterone (Figure 5d). Alternatively, there was not a decrease in *Crh* expression from 7 to 10 dpi in PLX5622‐treated tumor‐bearing animals, but rather an increase in expression, suggesting aberrant activation of *Crh*‐expressing neurons. We also observed increased expression of *Gnrh* (coding for gonadotropin‐releasing hormone) in PLX5622‐treated animals compared to AIN‐treated tumor‐bearing animals at 10 dpi (Figure 5d). There was no change in expression of *Kiss1*, which codes for kisspeptin, and peptide involved in gonadotropin‐releasing hormone regulation.

PLX5622 administration did not significantly affect astrogliosis in the MBH at 7 dpi, but did result in a significant decrease in GFAP signal intensity at 10 dpi (Figure S3a,c).

We also assessed whether PLX5622 administration resulted in neuronal loss in the MBH during PDAC. There was no change in NeuN+ neurons in the ARC of PLX5622‐treated tumor‐bearing animals compared to AIN‐treated tumor‐bearing animals (Figure S4).

### 3.7 PLX5622 administration does not alter systemic immune response during PDAC

**Figure 6:**
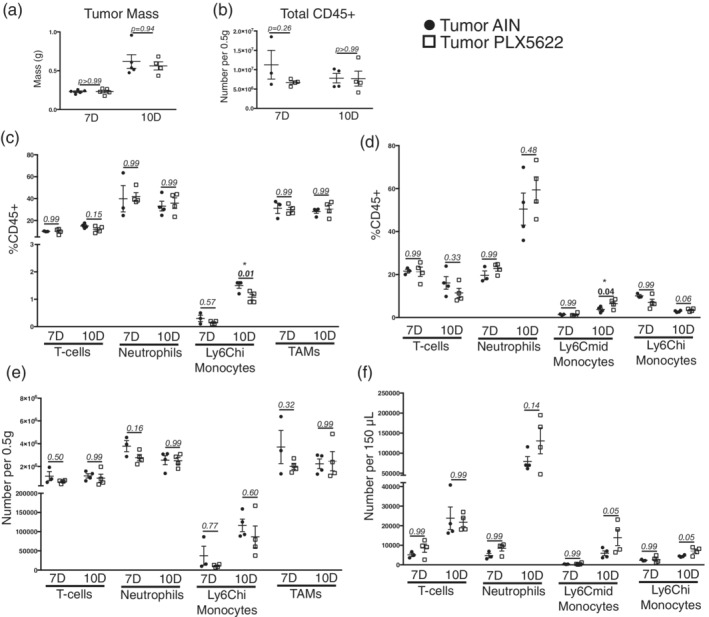
Minimal change in circulating and tumor‐associated immune cells after PLX5622 administration during PDAC. (a) Tumor mass in both AIN‐treated and PLX5622‐treated KPC‐bearing animals at 7 and 10 dpi. For all comparisons shown in this figure, *p* values reflect those from Bonferroni post‐hoc analysis in one‐way ANOVA analysis. (b) Total CD45+ cells in the tumors of AIN‐treated and PLX5622‐treated KPC‐bearing animals at 7 and 10 dpi. (c) Relative number of immune cells in the tumors of AIN‐treated and PLX5622‐treated KPC‐bearing animals at 7 and 10 dpi, expressed as a percentage of total CD45+ cells. (d) Relative number of immune cells in the blood of AIN‐treated and PLX5622‐treated KPC‐bearing animals at 7 and 10 dpi, expressed as a percentage of total CD45+ cells. (e) and (f) Total number of immune cells in the tumor (e) and blood (f) in AIN‐treated and PLX5622‐treated KPC‐bearing animals at 7 and 10 dpi *n* = 3–4/group

Since PLX5622 administration resulted in systemic CSF1‐R antagonism, and no studies have thoroughly assessed the effects of PLX5622 on macrophage populations outside the CNS, we tested whether this drug affected systemic immune response during PDAC. There was no change in tumor size at either 7 or 10 dpi in PLX5622‐treated animals (Figure 6a). We then used flow cytometry to quantify different immune cell populations in the blood, liver, and tumor in both PLX5622‐ and AIN‐treated PDAC‐bearing animals at 7 and 10 dpi. PLX5622 had minimal effect on circulating immune cells in the context of PDAC, as we observed no changes in absolute circulating immune cell number compared to AIN tumor animals at both 7 and 10 dpi (Figure S3 and 6f). There was only a small increase in Ly6C^hi^ monocytes and a small decrease in Ly6C^mid^ monocytes as a percentage of CD45+ cells in PLX5622‐treated animals at 10 dpi (Figure 6d). When comparing AIN‐treated to PLX‐treated tumor animals, we observed no differences in tumor‐infiltrating immune cells (Figure 6b,e), including T‐cells, neutrophils, monocytes, and tumor‐associated macrophages, both total number and as percentage of total CD45+ cells (Figure 6c,e).

**Figure 7:**
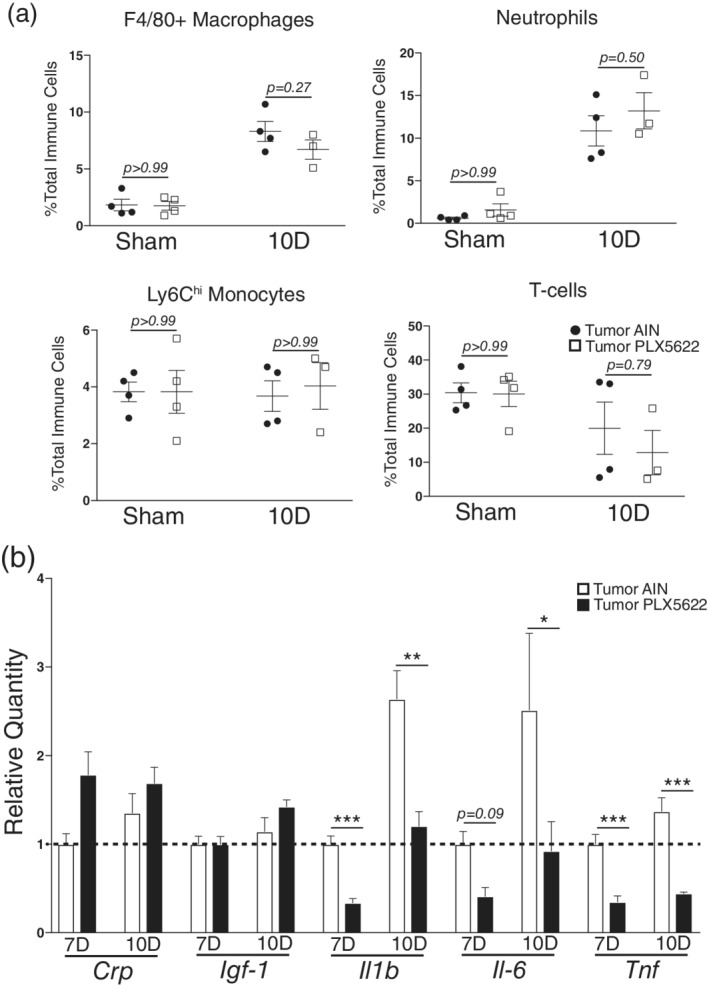
PLX5622 administration minimally effects liver inflammation during PDAC. (a) Quantification of different immune cell populations in the liver from sham animals and PDAC‐bearing animals. 10D = 10 days post inoculation. (b) qRT‐PCR of select inflammatory transcripts in the livers of PDAC‐bearing animals at 7 or 10 days post inoculation. For each transcript, values are normalized to that from 7 dpi. AIN tumor‐bearing group. ***p < .001, **p < .001 *p < .05 comparing AIN group to PLX56222 group in Bonferroni post hoc analysis in one‐way ANOVA. For all experiments, *n* = 3–4/group

Since liver inflammation was previously implicated in cachexia (Flint et al., 2016), we next investigated the effects of PLX5622 on the hepatic immune response during PDAC. We first used flow cytometry to determine whether PLX5622 altered the composition of immune cells in the liver. Since this is the first study to investigate the effects of PLX5622 on the liver, we chose to simplify analysis by only including sham animals and tumor animals at 10 dpi. While we were unable to compare absolute number of immune cells in the liver due to batch effects of flow cytometry, we observed no relative (as a percentage of total immune cells) changes in any immune cell population in the liver after PLX5622 administration during PDAC (Figures 7a and S6).

We next investigated the effects of PLX5622 administration on the expression of various inflammatory mediator transcripts in the liver throughout the course of PDAC cachexia. We analyzed expression of several transcripts associated with cachexia, including C‐reactive protein (*Crp*), insulin‐like growth factor 1 (*Igf‐1*), IL‐1ß (*Il‐1b*), IL‐6 (*Il‐6*), and TNF (*Tnf*) (Michaelis et al., 2017). We observed no differences in *Crp* or *Igf‐1* expression in PLX5622‐treated animals compared to sham animals at either 7 or 10 dpi. Alternatively, we observed a decrease in *Il‐1b* and *Tnf* in PLX5622‐treated animals at both 7 and 10 dpi and a decrease in *Il‐6* at 10 dpi (Figure 7b). Since it is unlikely that decreased expression of inflammatory cytokines in the liver resulted in increased cachexia, these results demonstrate that the effects of PLX5622 on anorexia, muscle catabolism, and fatigue during PDAC were not due to altered liver inflammation.

## 4 DISCUSSION

Previous studies implicated hypothalamic microglia as drivers of metabolic dysfunction during obesity, but none investigated the role of microglia in cachexia, a metabolic syndrome also driven by hypothalamic inflammation. We observed gliosis within the MBH throughout the course of PDAC cachexia. Microgliosis was specific to the MBH, while astrogliosis also occurred in the hippocampus. In vitro, PDAC‐derived factors induced microglia to produce arginase‐1, a potently anti‐inflammatory enzyme. Microglia depletion with an oral CSF‐1R antagonist worsened cachexia, including increased anorexia, fatigue, and muscle catabolism. Contrary to previous studies suggesting that microglia activation is detrimental in disease processes, these results implicate microglia as neuroprotective during pancreatic cancer cachexia.

We demonstrated that microglia in the MBH display an activated morphology early in PDAC cachexia, which correlated with astrogliosis in this region. Microgliosis peaked at 7 days post‐inoculation, a time point when animals were only beginning to develop anorexia and muscle catabolism. Alternatively, astrogliosis did not peak until 10 days post‐inoculation. This is the first study to show that microgliosis occurs during cancer cachexia. While reviews hypothesized that microglia activation may contribute to hypothalamic inflammation and subsequent symptoms of cancer cachexia (Molfino, Gioia, Rossi Fanelli, & Laviano, 2015), this was the first study to directly test this hypothesis. We observed that microglia assumed morphologies consistent with “activated microglia,” with retracted processes and increased soma size. One study by Norden et al., assessing the role of microglia in cancer‐associated fatigue and depression, reported that microglia in the cortex, but not the hippocampus, displayed an activated phenotype 3 weeks after inoculation with the colon‐26 tumor model (Norden et al., 2015). While we also did not observe microglia activation in the hippocampus, our results differ with those of Norden et al. in that we did not detect any differences in cortex microglia morphology in our PDAC tumor model. This discrepancy may be due to the tumor line (colon‐26 vs. KPC), mode of inoculation (subcutaneous vs. IP), or cancer stage (21 vs. 7/10 dpi). Moreover, the authors of the prior study did not report hypothalamic microglia morphology. Further studies are needed to assess neoplasm‐dependent alterations in microglia activation in distinct brain regions, and how these microglial populations contribute to cachexia symptoms.

We observed regional differences in microglia morphology throughout the brain, and even between different hypothalamic nuclei. While there were no differences in microglia morphology in the hippocampus and cortex of tumor‐bearing animals compared to sham animals, activated microglia were noticeable in the arcuate nucleus of tumor‐bearing animals as early as 7 dpi. Microglia activation was even more robust in the median eminence in tumor‐bearing animals. Studies investigating microglia activation in the context of HFDO showed similar results in that microglia in the MBH exhibited a more pronounced activated phenotype than in other brain regions (Baufeld, Osterloh, Prokop, Miller, & Heppner, 2016). Our results are likely due to the ME lacking a BBB and therefore providing free access to circulating factors during the progression of pancreatic cancer.

We observed an increased number of microglia, both in the arcuate nucleus and median eminence, early in PDAC cachexia. Many studies show that microglia accumulate in the MBH during HFDO (Baufeld et al., 2016; Thaler et al., 2012; Valdearcos et al., 2017), yet this is first study to show this phenomenon during a peripheral cancer. Previous studies demonstrated that monocytes infiltrate the MBH during HFDO and assume a microglia‐like morphology (Valdearcos et al., 2017). Interestingly, using a GFP BMT system that was similar to that incorporated in HFDO, we also observed infiltration of circulating immune cells in the MBH which expressed Iba‐1 and resembled macrophages. Future studies should assess the role of infiltrating macrophages in the MBH in PDAC to determine whether they are protective against cachexia.

Based on the hypothalamic microglia morphology we observed, indicative of an “activated status,” we hypothesized that microglia depletion via PLX5622 administration would attenuate cachexia. Surprisingly, PLX5622‐treatment worsened PDAC cachexia, suggesting that microglia are neuroprotective during pancreatic cancer. Our results are in agreement with a growing amount of literature showing that microglia activation is neuroprotective in a variety of contexts, including stroke (Otxoa‐de‐Amezaga et al., 2019), Alzheimer's disease (Hamelin et al., 2016), and multiple sclerosis (Napoli & Neumann, 2010). In contrast, our results differ from studies on anxiety, demonstrating that microglia activation is associated with anxiety‐induced impaired social interaction, which the authors used as one measure of sickness behaviors (Liu et al., 2018). We observed greatly decreased home cage locomotor activity in animals treated with PLX5622. These discrepancies suggest that microglia response to stimuli differs depending on context, and that inferring function based on morphology is not possible. This is further emphasized by the fact that, similar to PDAC cachexia, microglia assume an activated state in the MBH in HFDO, during which there is also induction of inflammatory cytokine transcripts, yet PLX5622 treatment ameliorated certain disease aspects, particularly food intake and body weight (Valdearcos et al., 2017). In contrast, we observed that PLX5622 treatment worsened all cachexia aspects that we measured, including anorexia, fatigue, and muscle catabolism. It is challenging to directly compare HFDO to PDAC cachexia, yet we observed that KPC‐conditioned media induces a completely different transcriptional response in microglia than that induced by saturated fatty acids (Valdearcos et al., 2014). KPC‐conditioned media induced *Arg1* and *Il‐6* (to a much lesser extent), while minimally inducing *Il‐1b* and even decreasing *Tnf* expression. These different results show that microglia in the MBH are able to respond to metabolic and inflammatory challenges in a context‐dependent manner. Furthermore, our results, along with those from HFDO studies, suggest that microglia function to preserve body mass during chronic systemic inflammation.

We observed increased HPA axis activation in PLX5622‐treated tumor‐bearing animals, which corresponded to increased expression of proteolytic transcripts in skeletal muscle and muscle catabolism. Our lab and others previously established a mechanism whereby hypothalamic inflammation causes activation of the HPA axis and glucocorticoid‐dependent muscle catabolism (Braun et al., 2011; Braun & Marks, 2015). In light of these findings, our current data suggest that microglia are important for HPA axis regulation during a peripheral cancer. Additional studies are needed to determine the nature of microglia‐neuronal interactions in the MBH during PDAC. Previous studies showed that microglia can cleave axons in the pituitary gland (Pow, Perry, Morris, & Gordon, 1989). Therefore, during PDAC, microglia may be important for cleaving neuronal projections in the hypothalamus, thereby modulating HPA‐axis activity.

We demonstrated, using an ex vivo culture system, that tumor‐derived factors stimulate microglia to produce arginase‐1 (Arg1), a peptidase that cleaves l‐arginine into urea and ornithine. PDAC is a powerful inducer of Arg1 in macrophages, likely from tumor‐derived lactate (Colegio et al., 2014). Arg1 is a potently anti‐inflammatory enzyme that modulates inflammation through directly competing with nitric oxide (NO) synthase for arginine (Chang, Liao, & Kuo, 1998). NO is a potently cytotoxic gaseous free radical that can cause neuronal damage and alter firing activity (Bossy‐Wetzel et al., 2004). Thus, it is conceivable that microglial induction of Arg1 is directly neuroprotective in the MBH, an area critical for regulation of food consumption and body mass. While we did not observe any difference in neuron number in the arcuate nucleus in PLX5622‐treated tumor‐bearing animals, future studies should assess markers of neuropathology in PLX5622‐treated tumor‐bearing animals (BBB breakdown, endoplasmic reticulum stress, reactive oxygen species generation, etc.) and determine if microglia can protect against NO‐mediated neuronal damage/dysfunction.

For microglia and other brain macrophages, Arg1 is commonly used as a polarization marker, indicating an “anti‐inflammatory” or “M2” phenotype (Orihuela et al., 2016). However, the function of resident macrophage‐derived Arg1 in the brain is unclear, and we believe judging function from polarization status is a gross oversimplification that can result in overlooking key functional features (Ransohoff, 2016). For example, we observed a robust decrease in *Il‐10* and *Tgfb* transcript in KPC‐conditioned media treated primary microglia. Both of these transcripts are frequently implicated as key “M2” transcripts, suggesting that during PDAC microglia function is far more complicated than a simple “pro‐inflammatory” or “anti‐inflammatory” phenotype. These results add to the growing amount of evidence that microglial polarization is not simply a dichotomous phenomenon, and the description of M1 or M2 phenotypes is an oversimplification of the complex biology of this cell type.

We observed astrogliosis in the MBH and hippocampus during PDAC. To our knowledge, this is the first study to demonstrate astrocyte activation in the brain during a peripheral cancer. Previous studies demonstrated astrogliosis in the spinal cord during PDAC (Saloman et al., 2016), and correlated this with muscle catabolism and fat loss in a mouse model of pancreatic cancer perineural invasion (Imoto et al., 2012). In contrast with microgliosis, astrocytosis was not specific to the MBH and also occurred in the hippocampus during PDAC. This may be due to the fact that astrocytes in the hippocampus send projections to the border of the third ventricle that form glial limitans and are directly in contact with the CSF. Alternatively, microglia in the hippocampus are not in direct contact with the CSF. We also observed increased astrogliosis in the MBH in PLX5622 treated animals. This suggests that microglia dampen astrocyte activation during PDAC. These results are in agreement with Gibson et al., which demonstrated that astrocyte activation and subsequent cognitive impairment could be ameliorated by microglia depletion in a model of chemotherapy‐induced neurotoxicity (Gibson et al., 2019). Additional studies are needed to determine the role of astrocytes in PDAC cachexia.

A few limitations should be considered when interpreting the results of the present study. We administered a systemic CSF‐1R antagonist, which may have exerted effects on macrophages outside the brain. Previous studies show that prolonged PLX5622 exposure can deplete tumor‐associated macrophages, but does not affect other macrophages populations, such as those in the spleen or marrow (Cavnar et al., 2013). However, we conducted an extensive analysis demonstrating that PLX5622 had little effect on macrophages outside the brain. Furthermore, while the focus of this manuscript was macrophages in the brain, other nervous system macrophages were likely affected by PLX5622 treatment. For example, microglia/macrophages in the spinal cord were previously shown to be activated in PDAC and therefore may contribute to decreased locomotor activity (Imoto et al., 2012). In addition, perineural invasion by tumor cells is associated with poor outcomes in PDAC, and is thought to be driven by endoneural macrophages (Cavel et al., 2012). Future studies should assess the effects of CSF1R antagonist on endoneural macrophages and subsequent perineural invasion. It is also worth noting that PLX5622 treatment can potentially have effects on microglia beyond “depletion,” which was suggested previously (Nissen, Thompson, West, & Tsirka, 2018). These effects are still not fully understood, but should be considered when interpreting the results of this manuscript.

We utilized a single model of PDAC cachexia during this study. While this model is extensively characterized and recapitulates most of the key features of cachexia observed in humans, future studies should utilize additional cachexia models to determine if this phenomenon is generalizable. Moreover, cachexia is induced relatively rapidly in our model (within 7 days of tumor cell inoculation), which may not accurately reflect the course of PDAC in humans. Lastly, PLX5622 administration may cause brain dysfunction. While it was previously reported that this was likely not the case (Elmore et al., 2014), and we demonstrated that PLX5622 treatment does not alter food intake, body weight, or home cage locomotor activity, future studies should utilize pharmacologic approaches to inhibit microglia activation to overcome this potential limitation.

In conclusion, microglia respond to tumor‐derived factors and assume an activated state in the MBH during PDAC. Depleting microglia or preventing their activation worsens cachexia, demonstrating their protective role during pancreatic cancer. Further studies are needed to identify the key protective molecular mediator presumably produced by these cells.

## Supporting information


**Figure S1** Astrogliosis occurs in the MBH and the hippocampus throughout PDAC cachexia. A) Representative confocal microscopy images GFAP immunoreactivity within the MBH and hippocampus from either a sham, 7 dpi, or 10 dpi mouse brain. Scale bar = 100 μm. B‐D) Analysis of GFAP fluorescent intensity (left) or percent area GFAP+ (right) in the arcuate nucleus, median eminence, or cortex. n.s. = not significant, **p < .01, *p < .05 compared to sham in one‐way ANOVA analysis. For all figures, bars depict mean ± *SEM*.

**Figure S2** PLX5622 administration does not alter food intake, body weight, or locomotor activity in the absence of PDAC. A) Cumulative food intake over six days prior to tumor implantation. B) Movement during wake cycle prior to tumor implantation, expressed as average counts per hour. C) Body mass as a percent of initial, measured six days after starting animals on either PLX5622 or AIN chow. n = 5‐9/group.

**Figure S3** PLX5622 depletes microglia in the MBH during PDAC. A) Representative 20X confocal microscopy images of the MBH from AIN‐ and PLX5622‐treated tumor‐bearing mice at 7 and 10 dpi. 3V = third ventricle. Scale bar = 50 μm. B) Quantification of Iba‐1+ microglia in the MBH. n = 3/group. ***p < .001 in one‐way ANOVA analysis comparing AIN to PLX5622 within 7 or 10 dpi group. C) Quantification of area occupied by GFAP immunoreactivity in the MBH at different stages of PDAC cachexia. n = 3/group. *p < .05 in one‐way ANOVA analysis comparing AIN to PLX5622 within 7 or 10 dpi group.

**Figure S4** No neuronal loss in the ARC during PDAC. A) Representative 20X images of NeuN immunofluorescence showing the MBH from AIN sham, AIN tumor, and PLX5622 tumor animals. ARC is outlined by dashed line. Results are from 10 dpi. Scale bar = 100 μm. B) Quantification of NeuN+ neurons in the ARC in AIN sham, AIN tumor, and PLX5622 tumor animals, 10 dpi. n = 4/group.

**Figure S5** Gating strategy for flow cytometry analysis of circulating and tumor‐infiltrating immune cells. Representative flow cytometry plots of gating strategies used to identify different immune cell populations in the blood (a) and tumor (b). TAMs = tumor associated macrophages.

**Figure S6** Gating strategy for flow cytometry analysis of circulating and tumor‐infiltrating immune cells. Representative flow cytometry plots of gating strategies used to identify different immune cell populations in the liver.

## Data Availability

The data that support the findings of this study are available from the corresponding author upon reasonable request.
