# Supplementary figures and images for: Microglia in the hypothalamus respond to tumor‐derived factors and are protective against cachexia during pancreatic cancer
Source: Glia. 2020 Feb 10;68(7):1479–94. doi: 10.1002/glia.23796 (PMC7205589; doi:10.1002/glia.23796)

**A**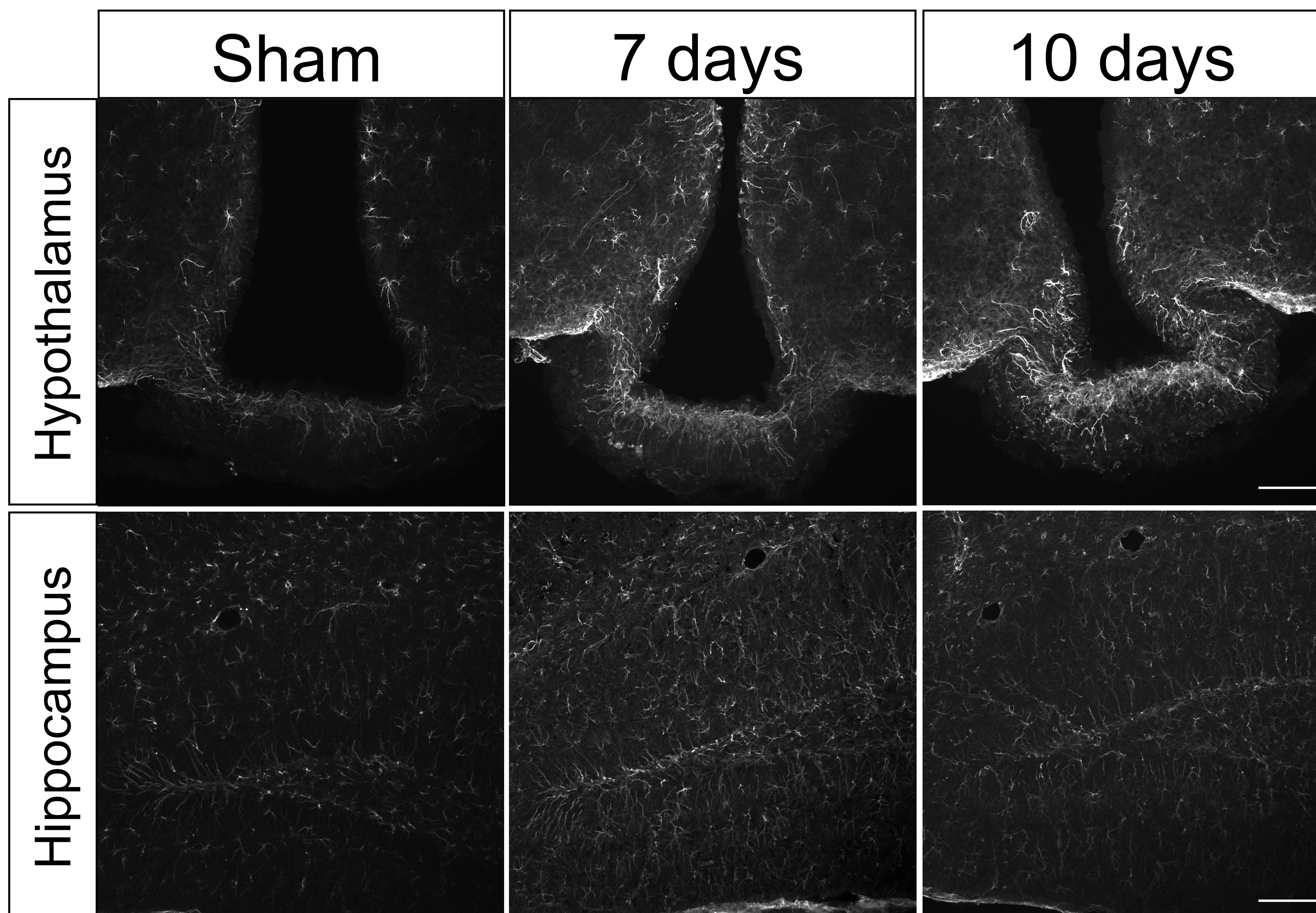**B****Arcuate Nucleus**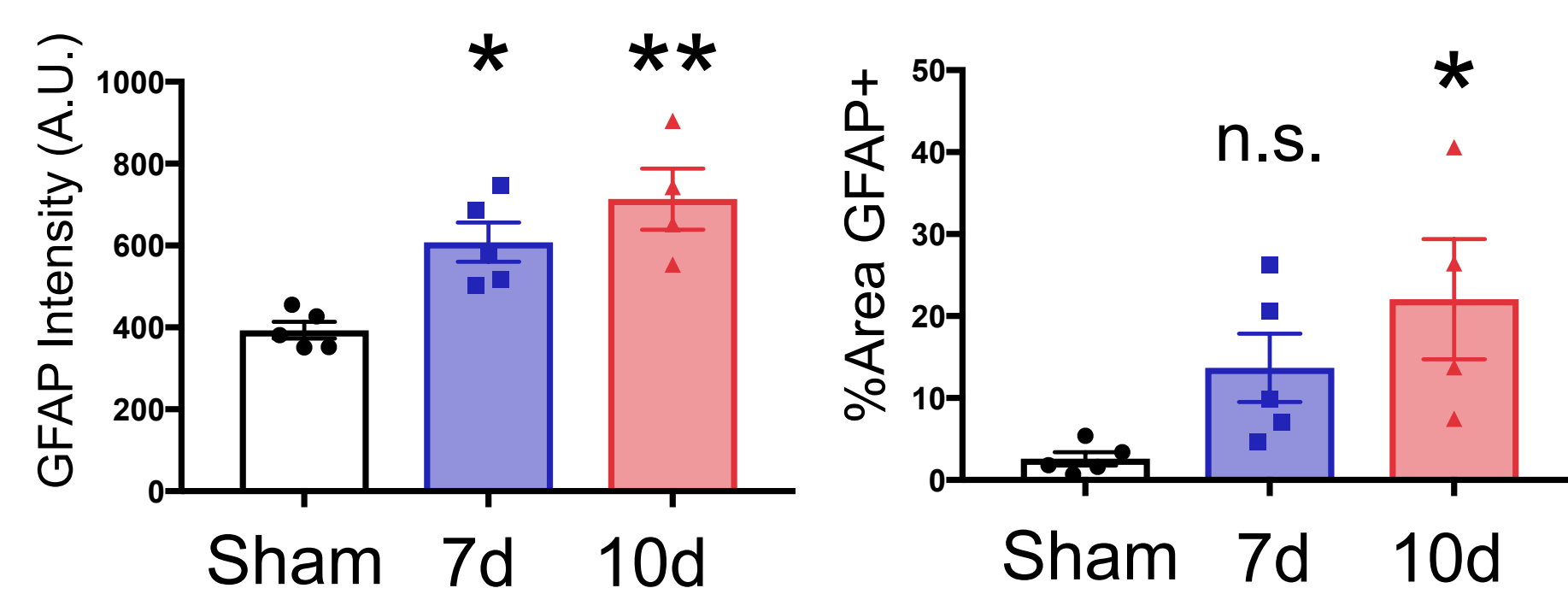**C****Median Eminence**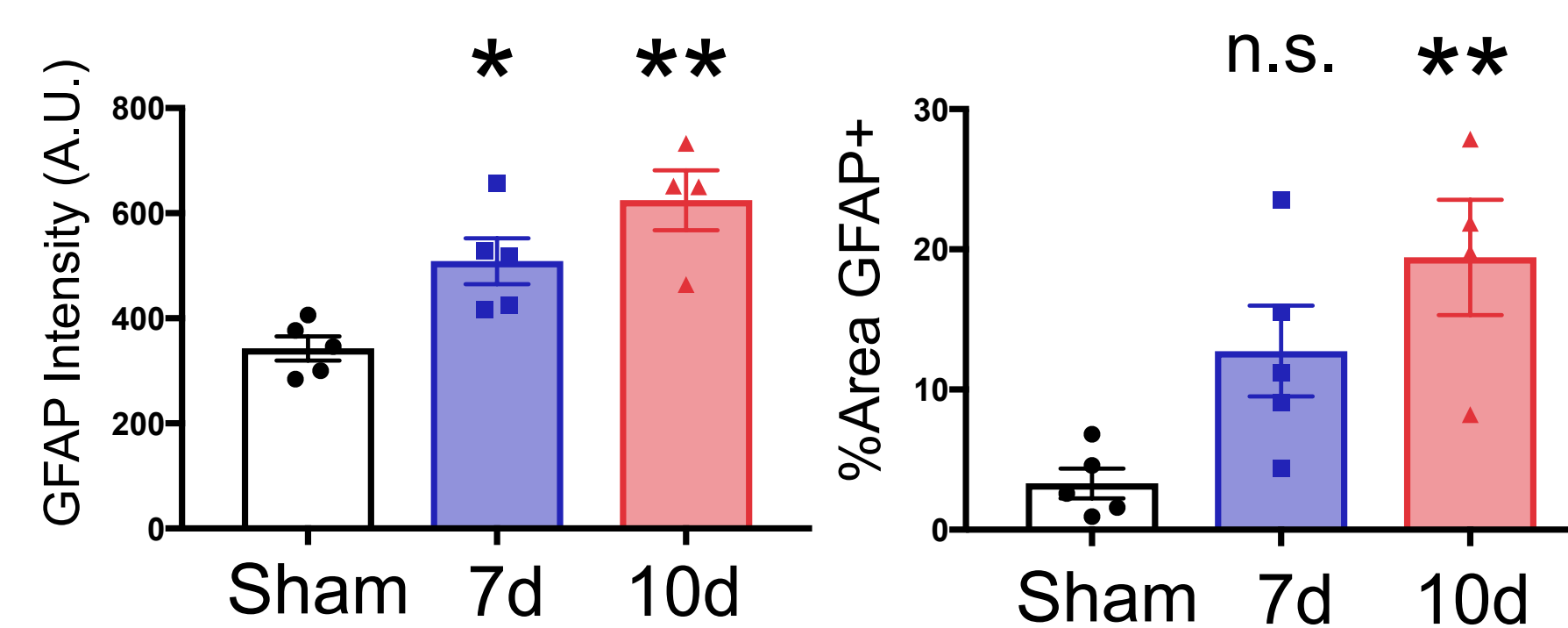**D****Hippocampus**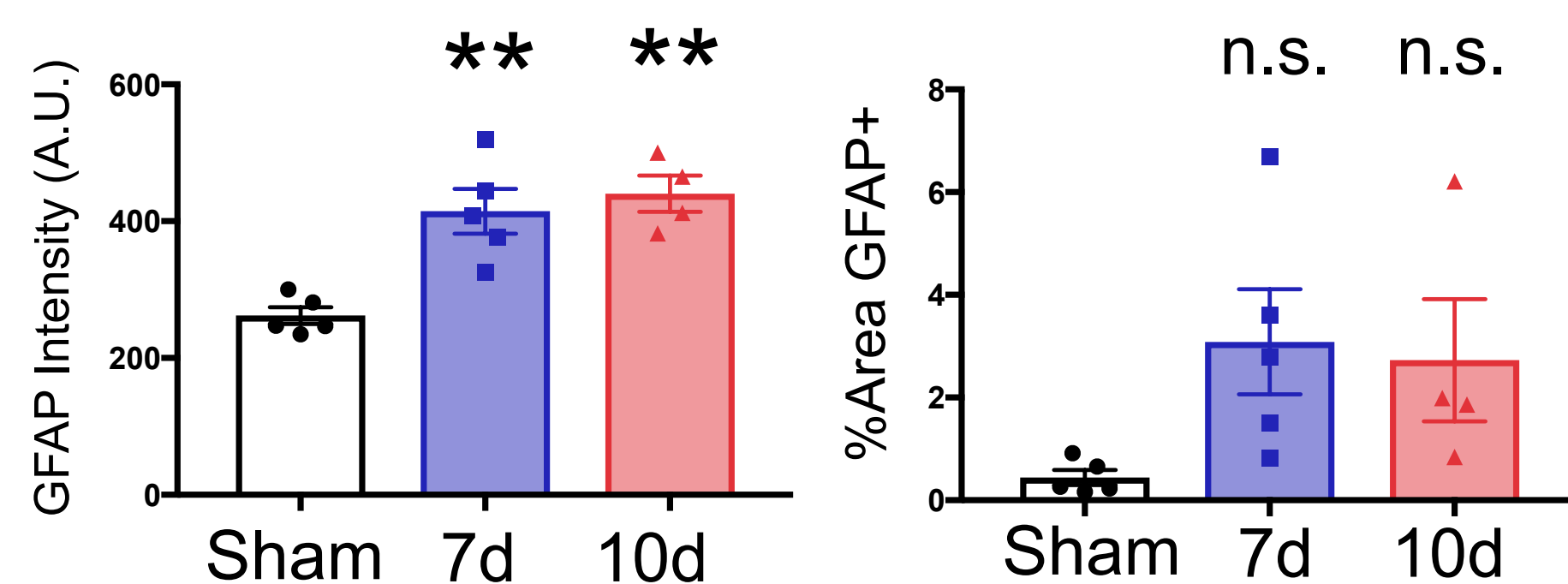

Supplement: Supplementary file 1 — Figure S1 Astrogliosis occurs in the MBH and the hippocampus throughout PDAC cachexia. A) Representative confocal microscopy images GFAP immunoreactivity within the MBH and hippocampus from either a sham, 7 dpi, or 10 dpi mouse brain. Scale bar = 100 μm. B‐D) Analysis of GFAP fluorescent intensity (left) or percent area GFAP+ (right) in the arcuate nucleus, median eminence, or cortex. n.s. = not significant, **p < .01, *p < .05 compared to sham in one‐way ANOVA analysis. For all figures, bars depict mean ± SEM. [file GLIA-68-1479-s001.pdf]

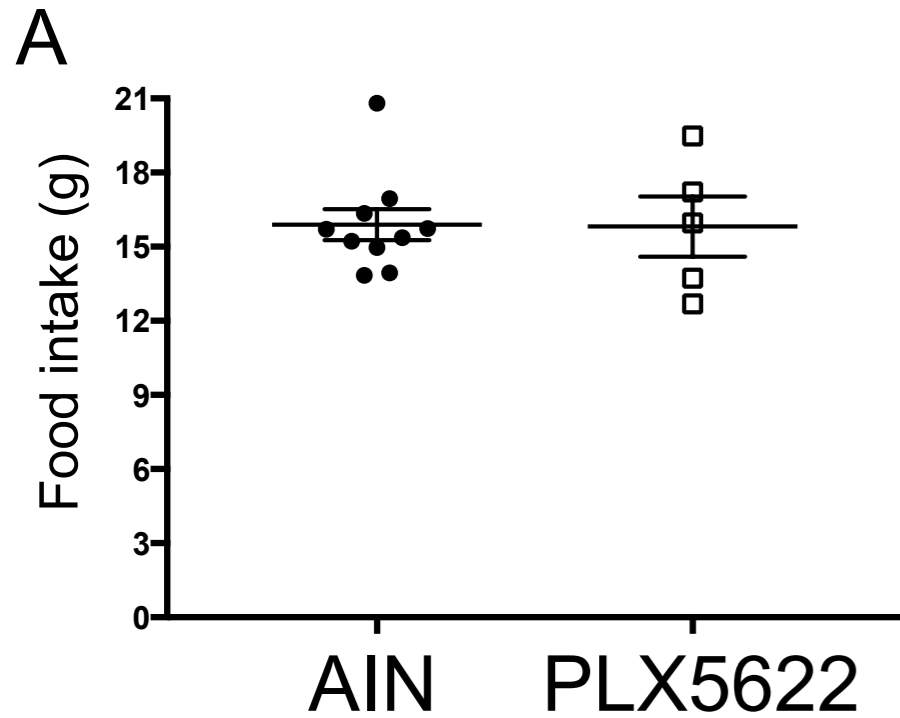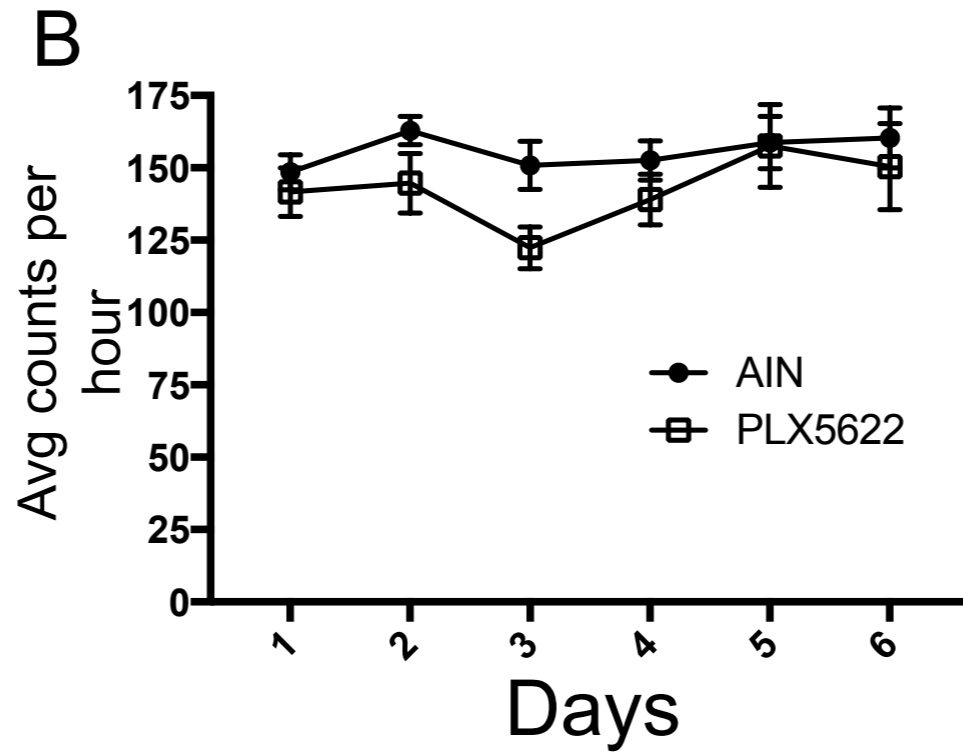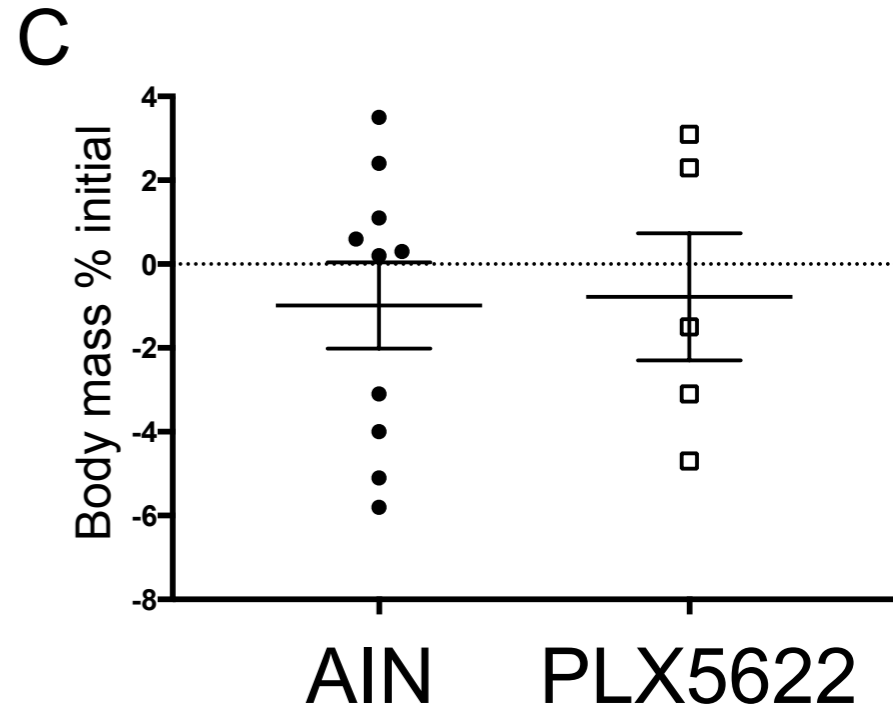

Supplement: Supplementary file 2 — Figure S2 PLX5622 administration does not alter food intake, body weight, or locomotor activity in the absence of PDAC. A) Cumulative food intake over six days prior to tumor implantation. B) Movement during wake cycle prior to tumor implantation, expressed as average counts per hour. C) Body mass as a percent of initial, measured six days after starting animals on either PLX5622 or AIN chow. n = 5‐9/group. [file GLIA-68-1479-s002.pdf]

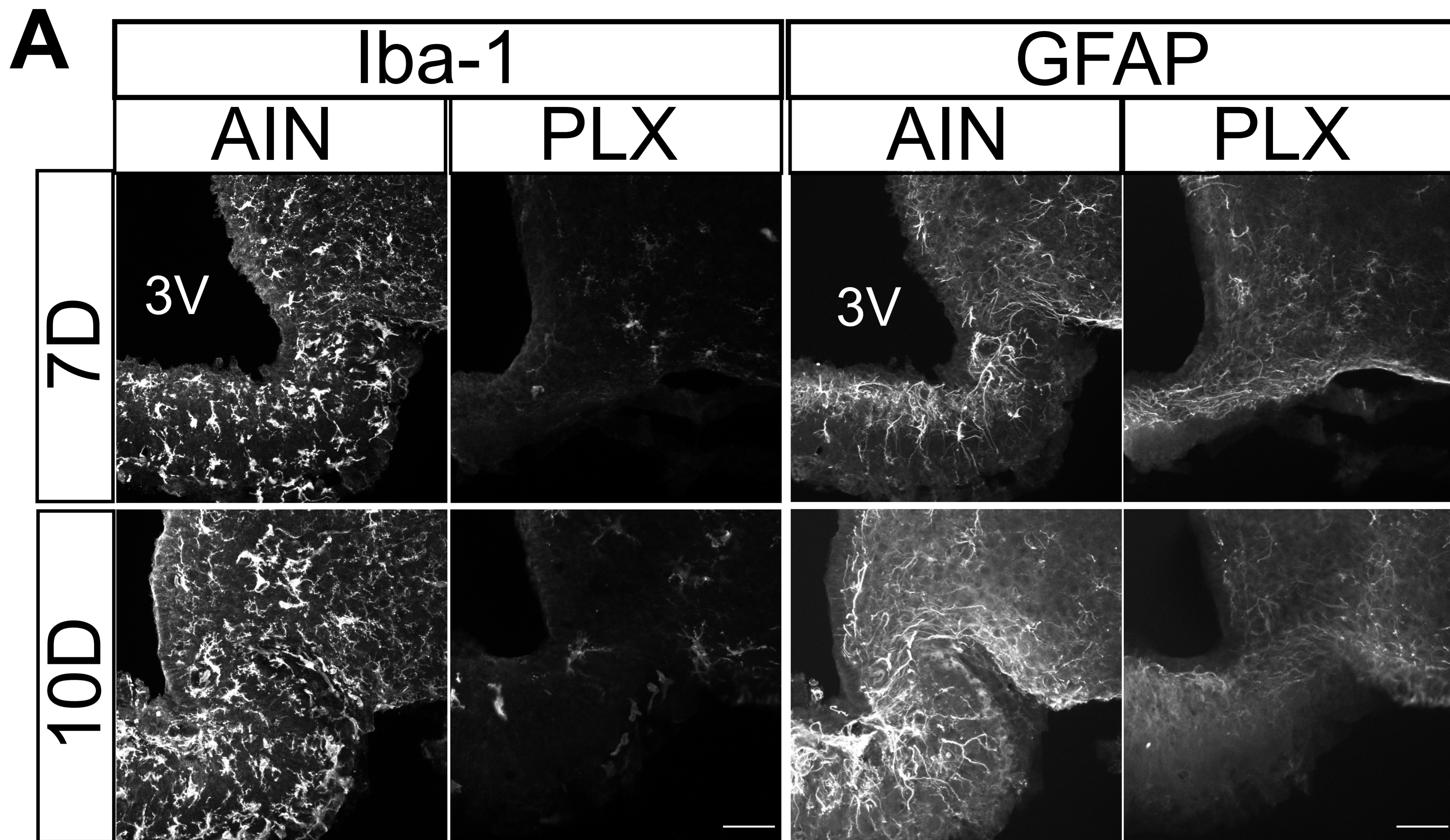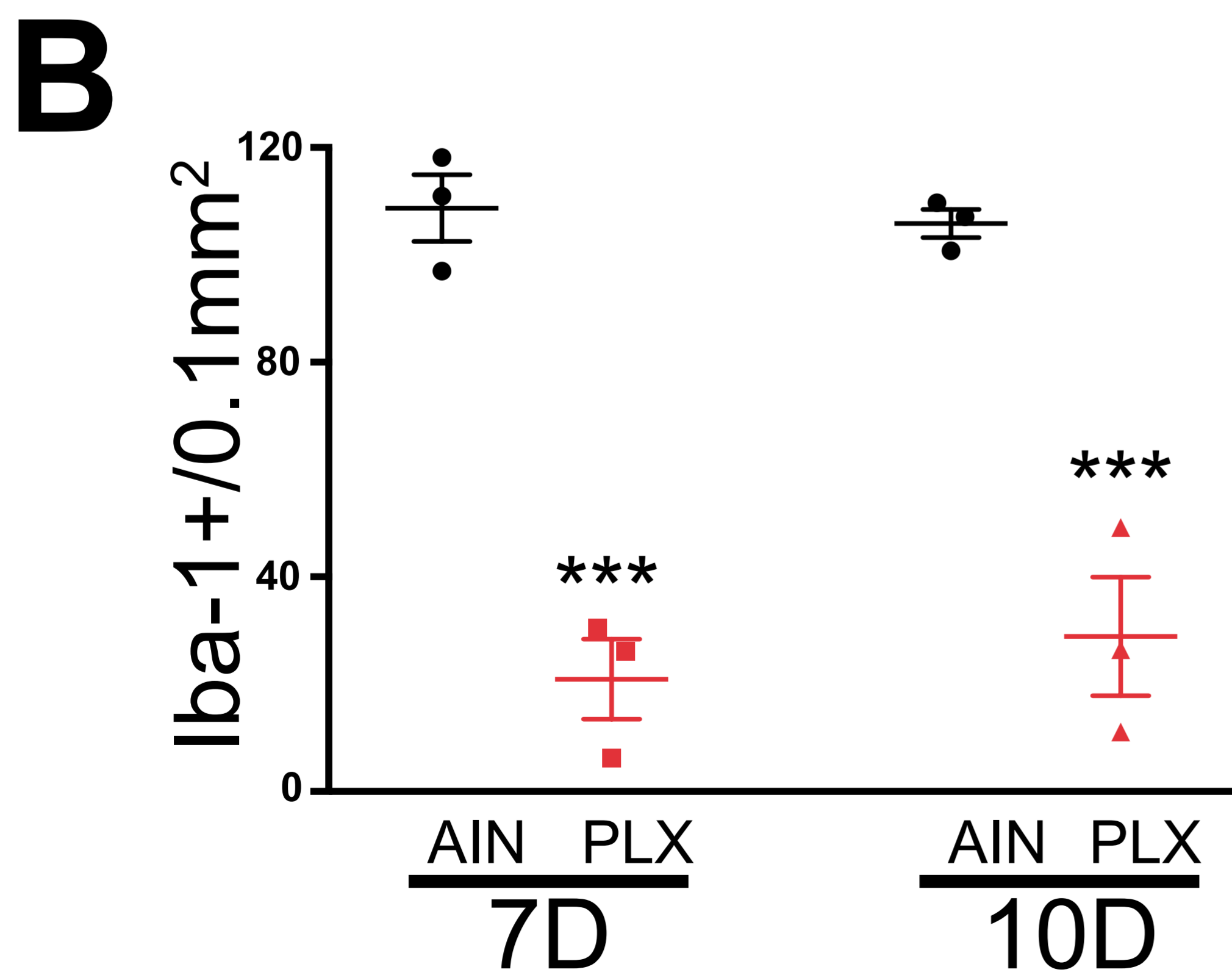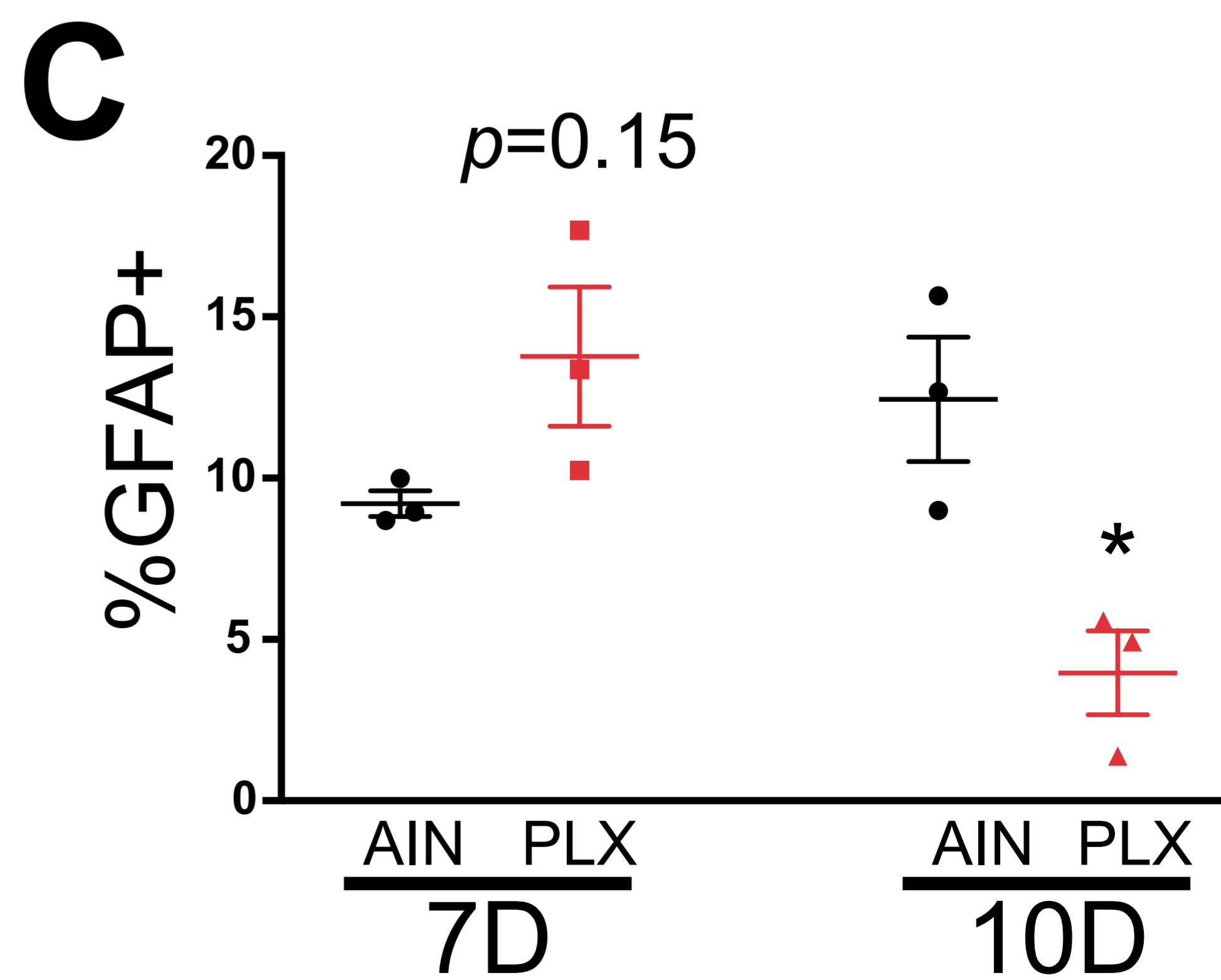

Supplement: Supplementary file 3 — Figure S3 PLX5622 depletes microglia in the MBH during PDAC. A) Representative 20X confocal microscopy images of the MBH from AIN‐ and PLX5622‐treated tumor‐bearing mice at 7 and 10 dpi. 3V = third ventricle. Scale bar = 50 μm. B) Quantification of Iba‐1+ microglia in the MBH. n = 3/group. ***p < .001 in one‐way ANOVA analysis comparing AIN to PLX5622 within 7 or 10 dpi group. C) Quantification of area occupied by GFAP immunoreactivity in the MBH at different stages of PDAC cachexia. n = 3/group. *p < .05 in one‐way ANOVA analysis comparing AIN to PLX5622 within 7 or 10 dpi group. [file GLIA-68-1479-s003.pdf]

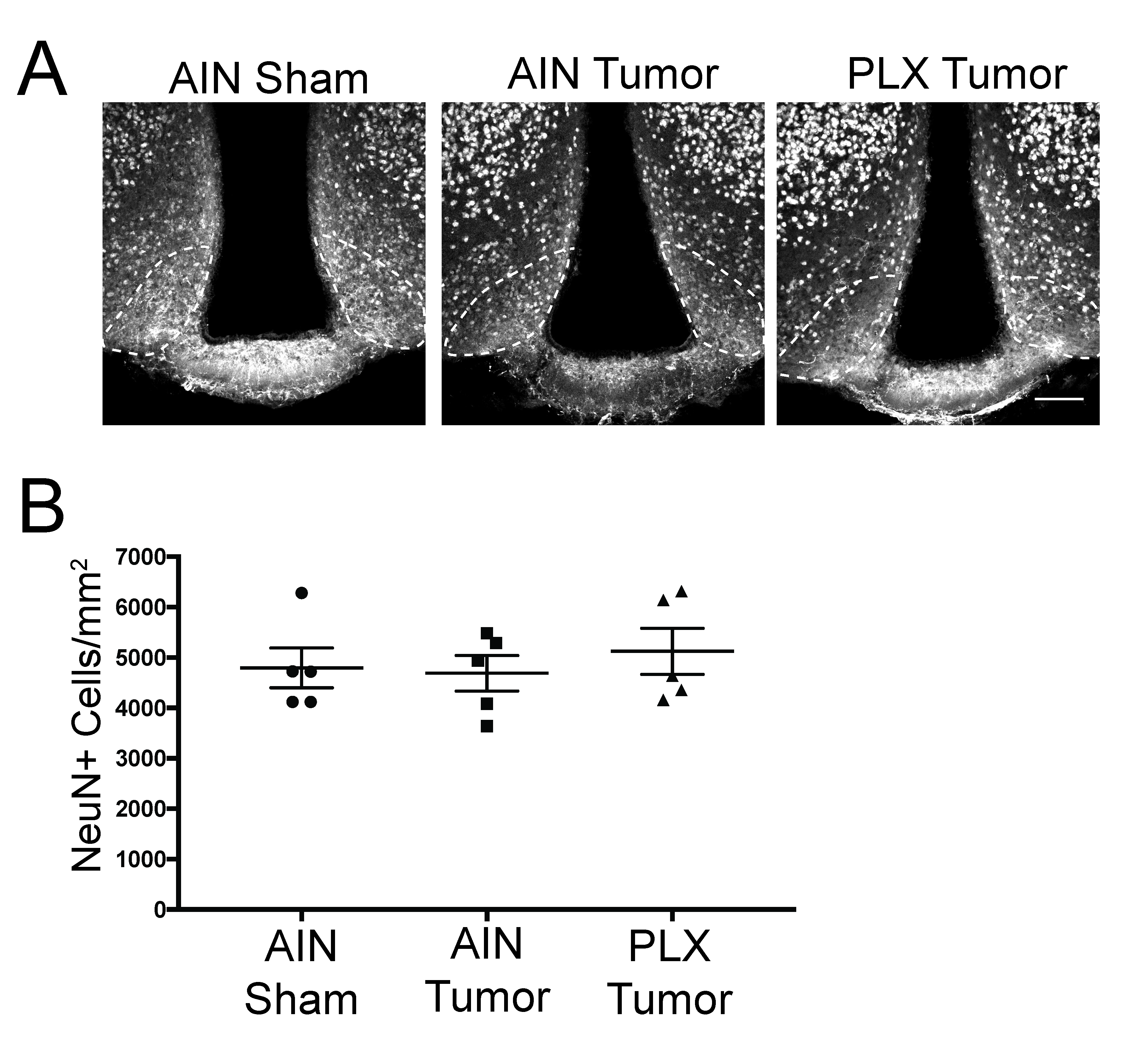

Supplement: Supplementary file 4 — Figure S4 No neuronal loss in the ARC during PDAC. A) Representative 20X images of NeuN immunofluorescence showing the MBH from AIN sham, AIN tumor, and PLX5622 tumor animals. ARC is outlined by dashed line. Results are from 10 dpi. Scale bar = 100 μm. B) Quantification of NeuN+ neurons in the ARC in AIN sham, AIN tumor, and PLX5622 tumor animals, 10 dpi. n = 4/group. [file GLIA-68-1479-s004.tif]

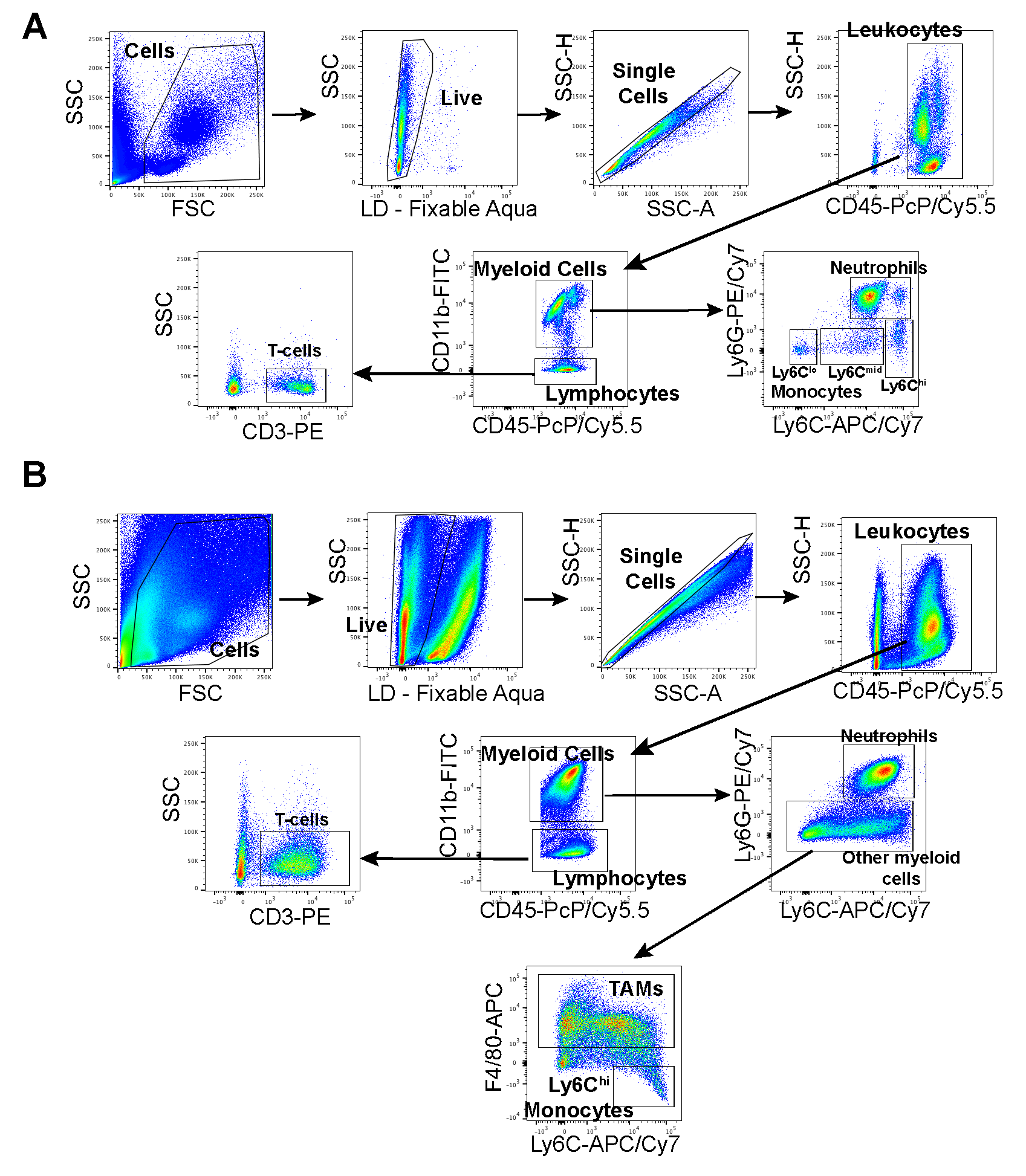

Supplement: Supplementary file 5 — Figure S5 Gating strategy for flow cytometry analysis of circulating and tumor‐infiltrating immune cells. Representative flow cytometry plots of gating strategies used to identify different immune cell populations in the blood (a) and tumor (b). TAMs = tumor associated macrophages. [file GLIA-68-1479-s005.tif]

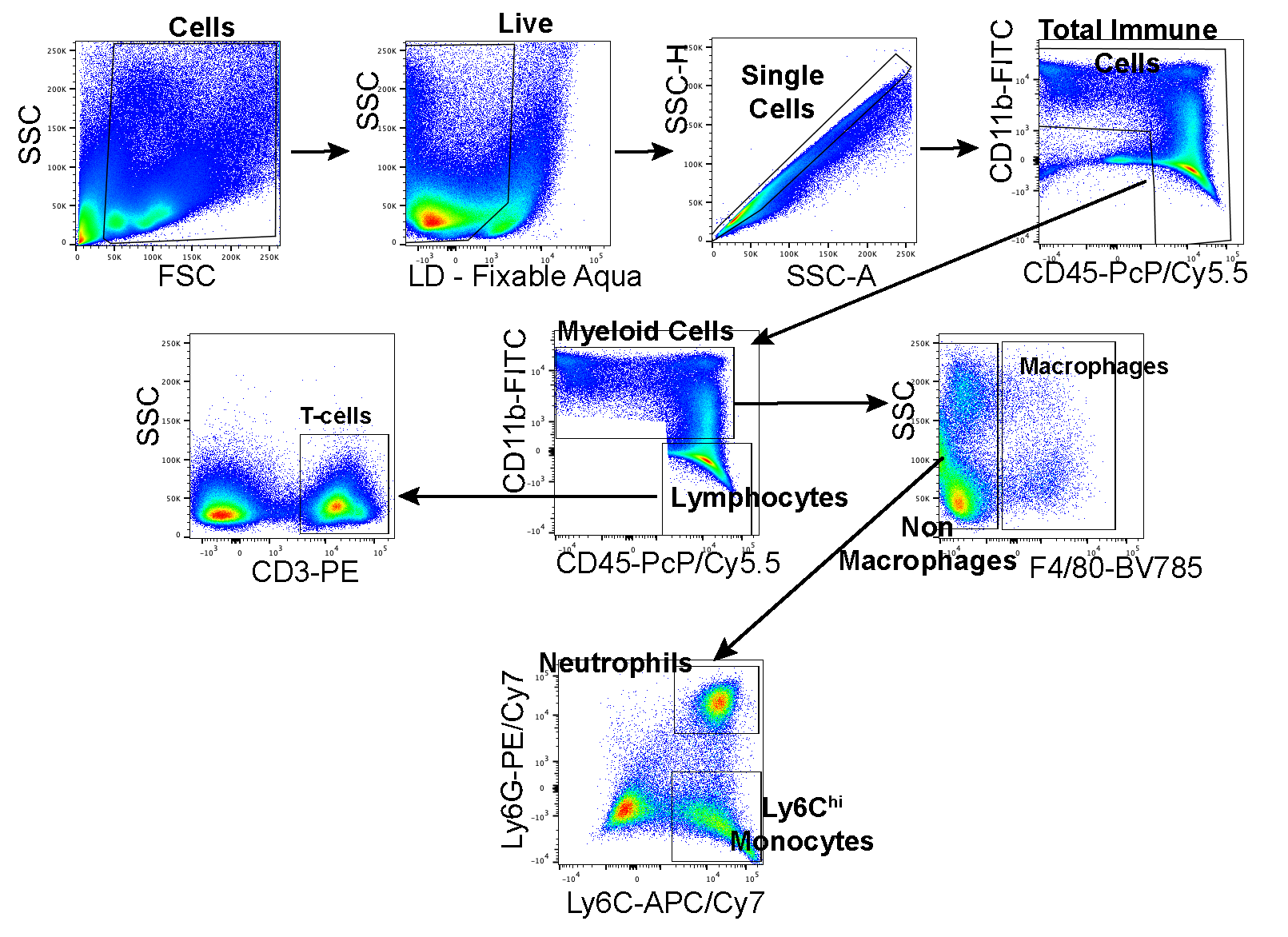

Supplement: Supplementary file 6 — Figure S6 Gating strategy for flow cytometry analysis of circulating and tumor‐infiltrating immune cells. Representative flow cytometry plots of gating strategies used to identify different immune cell populations in the liver. [file GLIA-68-1479-s006.tif]
